# Global regulation of mRNA translation and stability in the early *Drosophila* embryo by the Smaug RNA-binding protein

**DOI:** 10.1186/gb-2014-15-1-r4

**Published:** 2014-01-07

**Authors:** Linan Chen, Jason G Dumelie, Xiao Li, Matthew HK Cheng, Zhiyong Yang, John D Laver, Najeeb U Siddiqui, J Timothy Westwood, Quaid Morris, Howard D Lipshitz, Craig A Smibert

**Affiliations:** 1 Department of Molecular Genetics, University of Toronto, 1 King’s College Circle, Toronto, Ontario M5S 1A8, Canada; 2 Department of Biochemistry, University of Toronto, 1 King’s College Circle, Toronto, Ontario M5S 1A8, Canada; 3 Department of Cell & Systems Biology and Department of Biology, University of Toronto at Mississauga, 3359 Mississauga Road, Mississauga, Ontario L5L 1C6, Canada; 4 Banting and Best Department of Medical Research, Terrence Donnelly Centre for Cellular and Biomolecular Research, 160 College Street, Toronto, Ontario M5S 3E1, Canada; 5 Current address: Program in Developmental & Stem Cell Biology, Hospital for Sick Children Research Institute, Peter Gilgan Centre for Research and Learning, 686 Bay Street, Toronto, Ontario M5G 0A4, Canada

## Abstract

**Background:**

Smaug is an RNA-binding protein that induces the degradation and represses the translation of mRNAs in the early *Drosophila* embryo. Smaug has two identified direct target mRNAs that it differentially regulates: *nanos* and *Hsp83*. Smaug represses the translation of *nanos* mRNA but has only a modest effect on its stability, whereas it destabilizes *Hsp83* mRNA but has no detectable effect on *Hsp83* translation. Smaug is required to destabilize more than one thousand mRNAs in the early embryo, but whether these transcripts represent direct targets of Smaug is unclear and the extent of Smaug-mediated translational repression is unknown.

**Results:**

To gain a panoramic view of Smaug function in the early embryo, we identified mRNAs that are bound to Smaug using RNA co-immunoprecipitation followed by hybridization to DNA microarrays. We also identified mRNAs that are translationally repressed by Smaug using polysome gradients and microarrays. Comparison of the bound mRNAs to those that are translationally repressed by Smaug and those that require Smaug for their degradation suggests that a large fraction of Smaug’s target mRNAs are both translationally repressed and degraded by Smaug. Smaug directly regulates components of the TRiC/CCT chaperonin, the proteasome regulatory particle and lipid droplets, as well as many metabolic enzymes, including several glycolytic enzymes.

**Conclusions:**

Smaug plays a direct and global role in regulating the translation and stability of a large fraction of the mRNAs in the early *Drosophila* embryo, and has unanticipated functions in control of protein folding and degradation, lipid droplet function and metabolism.

## Background

Post-transcriptional regulatory mechanisms that function in the cytoplasm to control mRNA translation, stability and subcellular localization play essential roles in a wide variety of biological processes. While these types of controls function in a variety of cell types, they are particularly prevalent during early metazoan development where mRNAs synthesized from the mother’s genome direct the early stages of embryogenesis [1]. Indeed, genome-wide studies in *Drosophila*, *Caenorhabditis elegans*, zebrafish and mouse embryos have highlighted the substantial role that cytoplasmic post-transcriptional regulation plays in early embryos [1-13].

During early embryogenesis, regulation of specific transcripts is achieved through *cis*-acting elements that represent binding sites for microRNAs (miRNAs) or RNA-binding proteins. For example, miRNAs induce degradation of specific transcripts in both zebrafish and *Drosophila*[3,10]. Similarly the RNA-binding protein Smaug plays a major role in mRNA destabilization in the early *Drosophila* embryo [9]. Smaug is the founding member of a conserved family of post-transcriptional regulators that bind target mRNAs through stem-loop structures, known as Smaug recognition elements (SREs) [14-18]. SRE recognition by Smaug family members is mediated by a sterile alpha motif domain, which contains a cluster of conserved basic residues that functions as an RNA-binding surface [17,19-22].

Upon binding to target mRNAs Smaug family members repress translation and/or induce transcript decay through their ability to recruit various factors to a transcript [14-18,23,24]. For example, *Drosophila* Smaug can recruit the Cup protein to an mRNA and Cup in turn interacts with the cap-binding protein eIF4E [25]. The Cup-eIF4E interaction inhibits translation by blocking eIF4E-mediated 40S ribosomal subunit recruitment. Smaug can also recruit Argonaute 1 (AGO1) to an mRNA, thereby repressing translation [26]. Typically, Ago proteins are bound to small RNAs, such as miRNAs, that function to target the AGO1 protein to transcripts [27]. In contrast, Smaug can recruit AGO1 in a miRNA-independent manner [26].

Smaug can also remove an mRNA’s poly(A) tail through its ability to recruit the CCR4/NOT deadenylase [28-31]. In the case of at least one target mRNA this recruitment is thought to involve a complex containing Smaug and the Piwi-type AGO proteins Aubergine and AGO3 [32]. This complex has been proposed to bind this target transcript through SREs (bound by Smaug) together with sites complementary to piwi-RNAs (piRNAs) that are bound to AGO3 and/or Aubergine. Since the poly(A) tail plays a role in both initiating translation and stabilizing an mRNA, deadenylase recruitment can, in principle, both block translation and/or induce transcript decay.

Smaug has two well-characterized target mRNAs, *nanos* and *Hsp83*. Smaug represses *nanos* translation through two SREs in the *nanos* 3′ untranslated region (UTR) whereas loss of Smaug has only a modest effect on *nanos* mRNA stability [14-16,28,33]. In contrast, Smaug induces the degradation of *Hsp83* mRNA through eight SREs in the *Hsp83* open reading frame, while having no detectable effect on *Hsp83* translation [28,31]. Thus, Smaug can differentially regulate the expression of its target mRNAs.

*nanos* and *Hsp83* mRNAs are localized to the posterior of the embryo and Smaug’s regulation of these two transcripts is intimately associated with their localization. *nanos* mRNA is inefficiently localized to the posterior and *nanos* mRNA that escapes the localization machinery is found distributed throughout the bulk of the embryo where it is translationally repressed by Smaug [14-16,34,35]. *nanos* mRNA localized to the posterior is not repressed by Smaug and Nanos protein expression is thus restricted to the posterior of the embryo. *Hsp83* mRNA is uniformly distributed in early embryos and, as embryogenesis proceeds, Smaug degrades *Hsp83* mRNA in the bulk cytoplasm of the embryo while transcripts at the posterior of the embryo are protected [28,31,36,37]. This degradation/protection mechanism thus results in the localization of *Hsp83* mRNA to the posterior of the embryo.

In addition to *nanos* and *Hsp83* mRNA, Smaug is likely to regulate the expression of a large number of mRNAs in the early embryo through direct binding. For example, genome-wide experiments have shown that embryos collected from homozygous-mutant *smaug* females show stabilization of approximately 1,000 transcripts [9]. In addition, *smaug* mutant embryos also show cell-cycle defects associated with a failure of DNA replication checkpoint activation and they also fail to undergo zygotic genome activation [11,15]. As neither of these phenotypes can be explained by a defect in Smaug’s regulation of *nanos* or *Hsp83*, this is consistent with a role for Smaug in regulation of the expression of additional mRNAs.

To elucidate the global functions of Smaug in early embryos we employed two genome-wide approaches: 1) RNA co-immunoprecipitations followed by microarray analysis (RIP-Chip) to identify mRNAs that are bound by Smaug and 2) polysome gradients coupled to microarrays to identify targets of Smaug-mediated translational repression. Our data suggest that Smaug directly regulates the expression of a large number of mRNAs in the early embryo. Comparison of Smaug-bound mRNAs to those that are translationally repressed by Smaug (identified in this study), and those that are degraded in a Smaug-dependent manner [9] suggest that two-thirds to three-quarters of Smaug’s target mRNAs are either translationally repressed or degraded by Smaug. We also find that Smaug regulates the expression of multiple mRNAs that are localized to the posterior of the embryo. Gene set annotation enrichment analysis of the mRNAs directly bound by Smaug suggests that it regulates a diverse array of processes in the early embryo, including protein folding and degradation as well as metabolism. We present data indicating that Smaug regulates the expression of mRNAs encoding glycolytic enzymes (hexokinase and phosophofructokinase), a proteasome regulatory subunit (Rpn7) as well as epigenetic (Su(z)12) and post-transcriptional (Bicaudal C) regulators.

## Results

### The mRNAs encoded by 339 genes associate with Smaug

**Figure 1:**
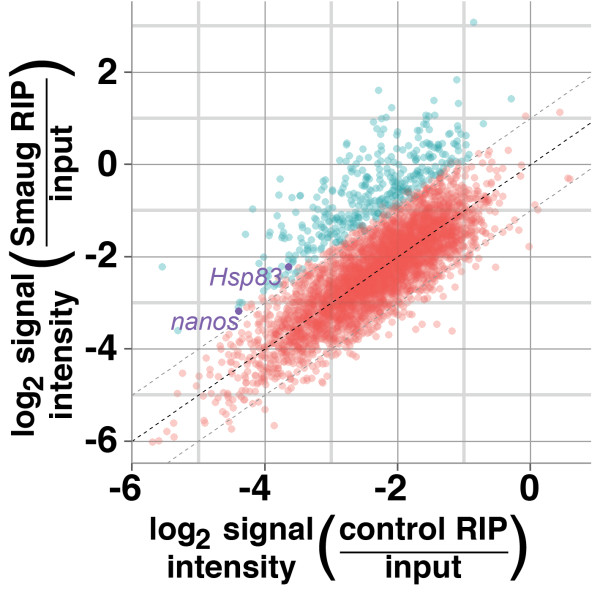
**Identification of Smaug-bound mRNAs.** The average, across three biological replicates and one technical replicate, of the microarray signal intensities of each expressed transcript in the Smaug and control RIPs divided by the signal intensities of each transcript in the immunoprecipitation inputs, were plotted against one another. SAM analysis allowed for the identification of 384 transcripts (blue dots) representing 339 genes that are enriched in the Smaug RIPs versus control RIPs at an FDR of <5%. The dots representing Smaug’s two known target mRNAs, *nanos* and *Hsp83*, are indicated. The dark dashed line represents no enrichment and the light dashed diagonal lines represent two-fold enrichment or depletion.

To identify Smaug’s target mRNAs on a genome-wide scale we used RIP-Chip. Extracts, prepared from 0- to 3-hour-old wild-type embryos, were immunoprecipitated with an anti-Smaug antibody (hereafter denoted as Smaug RIPs) while immunoprecipitations using non-immune serum served as a negative control (hereafter denoted as control RIPs). Genes that were not expressed or were expressed at low levels in starting crude extracts were removed from further analysis and Significance Analysis of Microarrays (SAM) [38] was then used to identify 339 genes whose mRNAs were significantly enriched in Smaug RIPs compared to control RIPs at a false discovery rate (FDR) of <5% (Figure 1; Additional files 1 and 2). Importantly, this list contains both of the well-characterized Smaug-target mRNAs, *nanos* and *Hsp83*.

**Figure 2:**
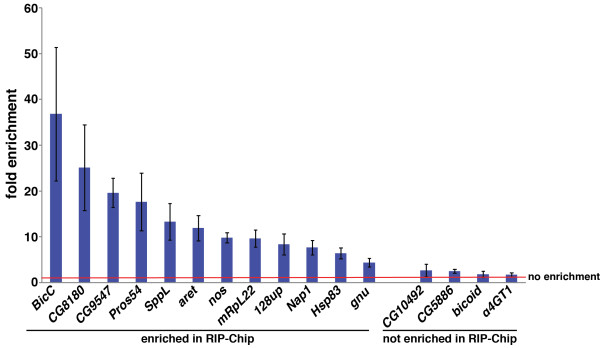
**Validation of Smaug-bound mRNAs.** The fold enrichment of mRNAs in Smaug RIPs versus control RIPs was determined via RT-qPCR and normalized to the levels of *RpL32* mRNA in the immunoprecipitated material. The red line indicates one-fold (that is, no) enrichment. Results are the average of three independent experiments and error bars indicate standard error of the mean.

To verify the quality of our microarray data we used reverse transcription followed by quantitative polymerase chain reaction (RT-qPCR) to assay the enrichment of specific mRNAs in Smaug RIPs compared to control RIPs. Twelve selected mRNAs from the RIP-Chip target list with FDRs <5%, including *nanos* and *Hsp83*, were enriched in Smaug RIPs compared to control RIPs. In contrast, four mRNAs that, based on our RIP-Chip data, are not bound by Smaug showed little or no enrichment (Figure 2).

### The mRNAs encoded by 342 genes are translationally repressed by Smaug

Smaug is a multifunctional regulator that is capable of both repressing translation and inducing the degradation of target mRNAs. To complement our identification of the targets of Smaug-mediated mRNA decay [9] and our identification of Smaug-bound mRNAs described above, we employed polysome gradients coupled with microarrays to identify targets of Smaug-mediated translational repression. This approach relies on the fact that the position of an mRNA in a polysome gradient is related to the number of ribosomes associated with that mRNA and can be employed to identify mRNAs that are regulated at the level of translation initiation [39-41]. As a first step towards applying this method we assessed the position of polysome-bound and free ribosomes in our gradients. Extracts prepared from 0- to 2-hour-old wild-type embryos were applied to polysome gradients in the absence or presence of EDTA. After centrifugation, gradients were separated into 12 equal fractions and the level of 18S rRNA in these fractions was determined via northern blot (Additional file 3). In the absence of EDTA, rRNA is distributed throughout the gradient, consistent with the presence of both free and polysome-associated ribosomes. In contrast, treatment with EDTA, which disrupts polysomes, resulted in a shift of 18S rRNA to the top fractions of the gradient. From these analyses we concluded that fractions 7 to 12 are exclusively polysomal, while fractions 5 to 6 are a mix of polysomal and non-polysomal material and fractions 1 to 4 are non-polysomal fractions. Subsequent gradients were, therefore, divided into four unequal pooled fractions, which, from the top to the bottom of the gradient were: pool 1 (fractions 1 to 4) containing free mRNAs; pool 2 (fractions 5 to 6) containing a mix of free and polysome-bound mRNAs; and pool 3 (fractions 7 to 9) and pool 4 (fractions 10 to 12), which both contain polysome-associated mRNAs.

RNA from the resulting pools was extracted and used to probe microarrays to assess the distribution of transcripts within the gradient. To quantify the level of translation for each gene we divided the average amount of the corresponding mRNA in pools 3 and 4 by the amount of mRNA in pool 1; and we define the translation index (TI) as the log_2_-transformed version of this ratio. We removed genes from the polysome data that were not expressed or were expressed at only low levels. We also omitted the data from pool 2 in the TI calculation as it represents a mixed population of translated and translationally repressed mRNAs. We note that inclusion of pool 2 in the TI calculation has little effect on the calculated TI (Additional file 4).

**Figure 3:**
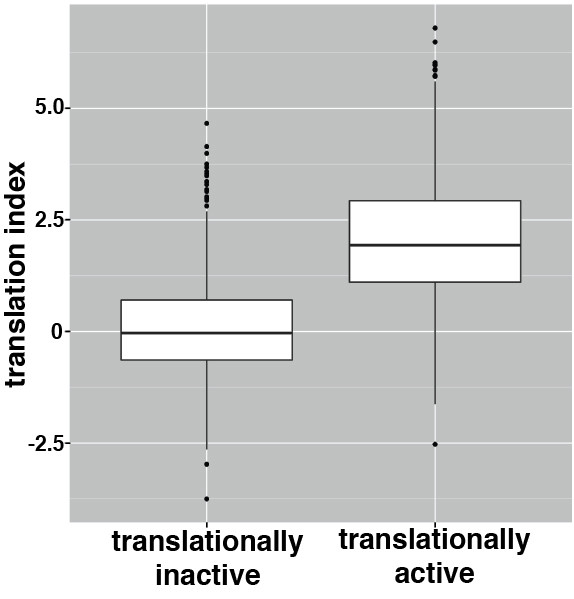
**Validation of polysome-gradient microarrays.** TIs calculated in this study were used to generate box plots to compare the range of TIs for genes that were previously categorized in Qin *et al*. [8] as ‘translationally active’ or ‘translationally inactive’ in embryos from the same developmental stage.

We then compared the TI for each gene in wild-type embryos to previously published polysome/microarray data from similarly staged wild-type embryos [8]. In that previous study mRNA levels were assayed across polysome gradients divided into 12 fractions and genes whose mRNAs were preferentially translated or preferentially untranslated were identified. Figure 3 shows that the TI calculated from our data is significantly higher for the preferentially translated group of mRNAs compared to the preferentially untranslated group (Wilcoxon rank-sum test, *P* < 3 × 10^-16^), indicating an excellent correlation between the two data sets.

**Figure 4:**
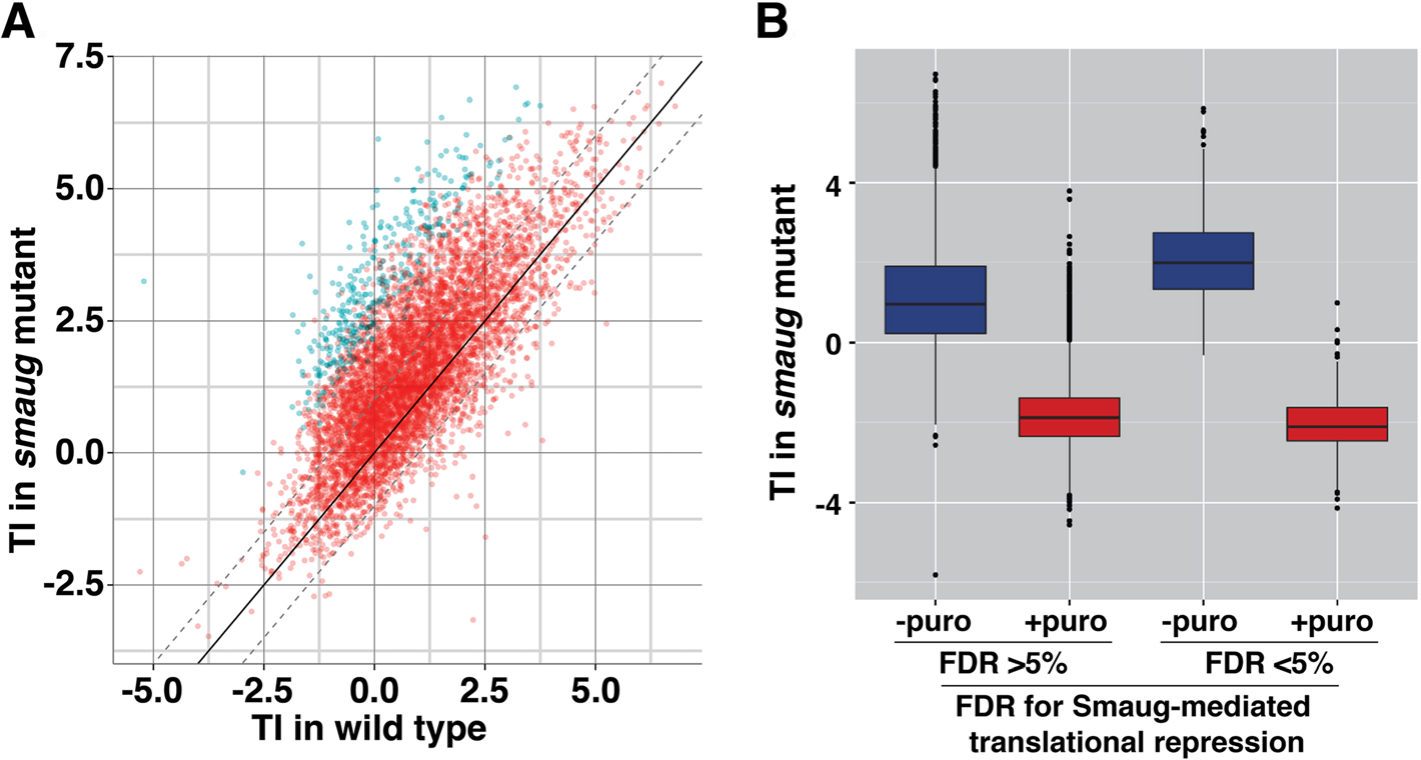
**Identification of the targets of Smaug-mediated translational repression. (A)** The averages, across three biological replicates, of the TI in *smaug*-mutant and wild-type embryos were plotted against one another. SAM analysis allowed for the identification of 359 transcripts (blue dots) representing 342 genes that show an increase in TI in *smaug* mutant versus wild type at an FDR of <5%, while the red dots represent genes with FDRs of >5%. The solid diagonal line represents no enrichment and the dotted diagonal lines represent two-fold enrichment or depletion. **(B)** Polysome gradients from *smaug*-mutant embryos were performed with or without puromycin treatment and the average, across two biological replicates, of the TI for each gene was calculated. Box plots show the range of TIs for genes where the TI increased in *smaug*-mutant embryos versus wild type with an FDR <5% and those with an FDR >5%, as defined in **(A)**.

To identify mRNAs that are translationally repressed by Smaug, we fractionated extracts from embryos collected from 0- to 2-hour old homozygous mutant *smaug* mothers (hereafter denoted as ‘*smaug*-mutant embryos’). We then compared the TI for each expressed gene in wild-type and *smaug*-mutant embryos (Figure 4A; as above, we note that inclusion of pool 2 in the TI calculation has little effect on the calculated TI, see Additional file 5). We expected the mRNA targets of Smaug-mediated translational repression to shift their distribution from pool 1 in wild-type embryos to pools 3 and 4 in *smaug* mutant embryos, thus resulting in an increase in those genes' TIs. Using SAM we identified 342 genes, with an FDR of <5%, where the TI increased in *smaug*-mutant embryos versus wild type (Figure 4A; Additional files 6 and 7). These genes represent a high-confidence list of Smaug-mediated translational repression targets. As expected, neither *Hsp83* nor *nanos* mRNA was present in this high-confidence list: first, using metabolic labeling, we previously showed that Smaug has no effect on *Hsp83* translation [28]; second, Clark *et al*. [42] have shown that a substantial fraction of translationally repressed *nanos* mRNA is associated with polysomes, consistent with our observation that approximately 54% of *nanos* mRNA is polysome-associated in wild-type embryos.

### Targets of Smaug-mediated translational repression are recruited to polysomes in a *smaug* mutant

To confirm that the increase in TI was indeed the result of the recruitment of mRNAs onto polysomes, *smaug*-mutant extracts were treated with puromycin, applied to polysome gradients and the resulting fractions were then analyzed via microarray. Puromycin is a translational inhibitor that causes premature chain termination during translation, thereby releasing mRNAs from polysomes. Figure 4B shows that puromycin causes a significant decrease in the TI (Fisher’s exact test, *P* < 3 × 10^-16^) for the bulk of mRNAs present in *smaug*-mutant embryos (that is, those genes whose mRNAs show a FDR >5%), consistent with the fact that the majority of the mRNAs that are present in pools 3 and 4 of our gradients are indeed polysome-associated. Similarly, we also saw a significant decrease in the TI (Fisher’s exact test, *P* < 3 × 10^-16^) for the 342 genes that are targets of Smaug translational repression (FDR <5%), consistent with the fact that, in *smaug*-mutant embryos, these mRNAs are highly associated with polysomes.

### Smaug is likely to repress the translation of approximately 3,000 mRNA targets

**Figure 5:**
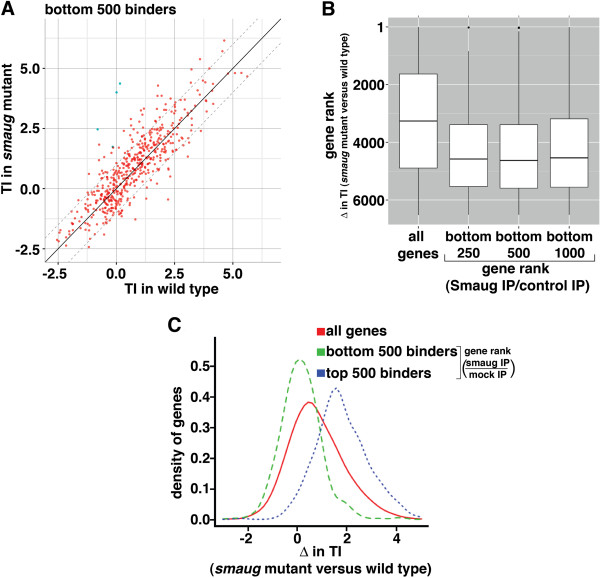
**Smaug represses the translation of thousands of mRNAs in the early embryo. (A)** The 500 bottom Smaug binders are the 500 genes whose mRNAs show the lowest fold enrichment in Smaug RIPs versus control RIPs and they were plotted as in Figure 4A. The solid diagonal line represents no enrichment and the dotted diagonal lines represent two-fold enrichment or depletion. **(B)** Genes were ranked based on the extent of the increase in TI in *smaug* mutant versus wild type, with the gene having the highest increase being ranked number one. Box plots were then used to show the range of ranks for all genes, and the bottom 250, 500 and 1,000 Smaug binders as defined in **(A)**. **(C)** Kernel density plot showing the change in TI in *smaug* mutant versus wild type for the bottom 500 Smaug binders as defined in **(A)** compared to the top 500 Smaug binders and all genes in the data set.

In addition to those genes that meet an FDR of <5% (shown in blue in Figure 4A) the TI of a large number of additional genes increased in *smaug* mutants. This suggests that a substantial subset of the genes with >5% FDR are potential targets of Smaug-mediated translational repression. Since SAM corrects for an average change in TI, if a large proportion of transcripts were in fact translationally repressed by Smaug, SAM would over-correct, thereby increasing the number of false negatives. To further evaluate the extent of Smaug-mediated translational repression we generated lists of genes that encode mRNAs that are unlikely to be bound by Smaug and are, therefore, unlikely to be targets of Smaug-mediated translational repression and then assessed their behavior in the polysome-gradient microarray experiments. We did this by identifying the 250, 500 and 1,000 genes whose mRNAs showed the lowest fold-enrichment in Smaug RIPs versus control RIPs. A comparison of the TI for each of these genes in wild-type and *smaug*-mutant embryos showed a distribution with little bias towards an increase in TI in the *smaug* mutant, confirming that few are likely to be targets of Smaug-mediated translational repression (Figure 5A; Additional file 8). In general, most genes not bound by Smaug had TI changes below the median of the *smaug* mutant (see Figure 5B where genes were ranked based on the extent of the increase in TI in *smaug*-mutant versus wild type, with the gene having the highest increase being ranked number one). This trend is highly significant (for example, 350 of the 500 ‘unbound’ list are below the median and the distributions of the bottom 250, 500 and 1,000 genes are all significantly different from the distribution for all genes; Fisher’s exact test, *P* < 3 × 10^-16^).

Finally, we performed a kernel density estimation of the change in TI for the genes whose mRNAs fell into the top 250, 500 and 1,000 Smaug-bound transcripts (that is, those mRNAs with the highest fold-enrichment in Smaug RIPs compared to control RIPs) as compared with the 250, 500 and 1,000 genes whose mRNAs were unlikely to be bound by Smaug (that is, with the lowest fold-enrichment in Smaug RIPs versus control RIPs). This analysis showed a peak change of TI in *smaug*-mutant embryos versus wild type of 1.57, 1.49 and 1.49 (linear fold-change of 2.97, 2.80 and 2.80) for each of the top three sets of bound transcripts, respectively (Figure 5C; Additional file 9). In contrast, for each of the unbound sets there was a peak TI change of only −0.01, 0.10, and 0.12 (linear fold-change of 0.99, 1.07, and 1.09), respectively (Figure 5C; Additional file 9). The fact that transcripts not bound by Smaug had no change in TI, on average, suggests that our TI estimates are directly comparable between the *smaug*-mutant and wild-type datasets. As such, the distribution of TI changes for all genes is consistent with Smaug repressing the translation of a large number of mRNAs in the early *Drosophila* embryo.

To estimate the actual number of genes that are translationally repressed by Smaug, we deconvolved the distribution of TI changes for all genes (Figure 5C; Additional file 9) to estimate the relative contributions of genes whose TI changes are distributed according to the top *N* and bottom *N* Smaug-binders (for N = 250, 500, and 1,000), respectively. Based on this analysis, we estimated that 3,135, 3,094, or 2,728 are likely to be translationally repressed by Smaug using the distributions for *N* = 250, 500, or 1,000, respectively (for details see Materials and methods). We conclude that Smaug represses the translation of approximately 3,000 mRNAs in early embryos, representing about half of the 5,886 genes whose expression we detected in the polysome-microarray data set.

### SRE stem-loops are highly enriched in Smaug’s target mRNAs

Smaug binds to and regulates its target mRNAs through SRE stem-loop structures and, as such, we would expect that mRNAs bound by Smaug as well as mRNAs translationally repressed by Smaug would be enriched for these stem-loops. The consensus sequence for the SRE loop is CNGGN_0-3_ (where N is any base) [17,20]. The variability in the number of nucleotides at the 3′ end of the loop derives from structural studies showing that while the RNA-binding domain of the yeast Smaug homolog, Vts1p, interacts with the loop and stem 5′ to the loop, it does not make contact with the 3′ region of the loop [20,22]. Thus, loop sequences where N is greater than 3 at this position are also expected to be Smaug-binding sites.

To ask whether SREs are predictive of Smaug binding and translational repression we searched all expressed genes in the RIP-Chip and polysome-microarray datasets for stem-loops with the loop sequence CNGGN_0-4_ (see Materials and methods for details). Our method assigned a probability for each potential SRE within a transcript based on the likelihood that it would fold into a stem-loop structure where the loop matches the CNGGN_0-4_ consensus. For each mRNA, an SRE score was then calculated as the sum of the probabilities for each SRE within that mRNA [43]. Strikingly, for the RIP-Chip experiment, bound mRNAs (FDR <5%) had a median SRE score of 25.9 whereas unbound mRNAs (FDR >5%) had a 10-fold lower SRE score (2.4). Likewise, for the polysome-microarray experiment, repressed mRNAs (FDR <5%) had a median SRE score of 36.2 whereas unrepressed mRNAs (FDR >5%) had a median SRE score of only 3.9. Within each of the regulated sets, however, the mRNAs nearer the top of the list (top 50 or top 100 as defined by fold-enrichment in Smaug RIPs versus control RIPs for binding or the change in TI between *smaug*-mutant and wild type for translational repression) did not have higher SRE scores than the median for the bound or repressed mRNAs with FDR <5%.

**Figure 6:**
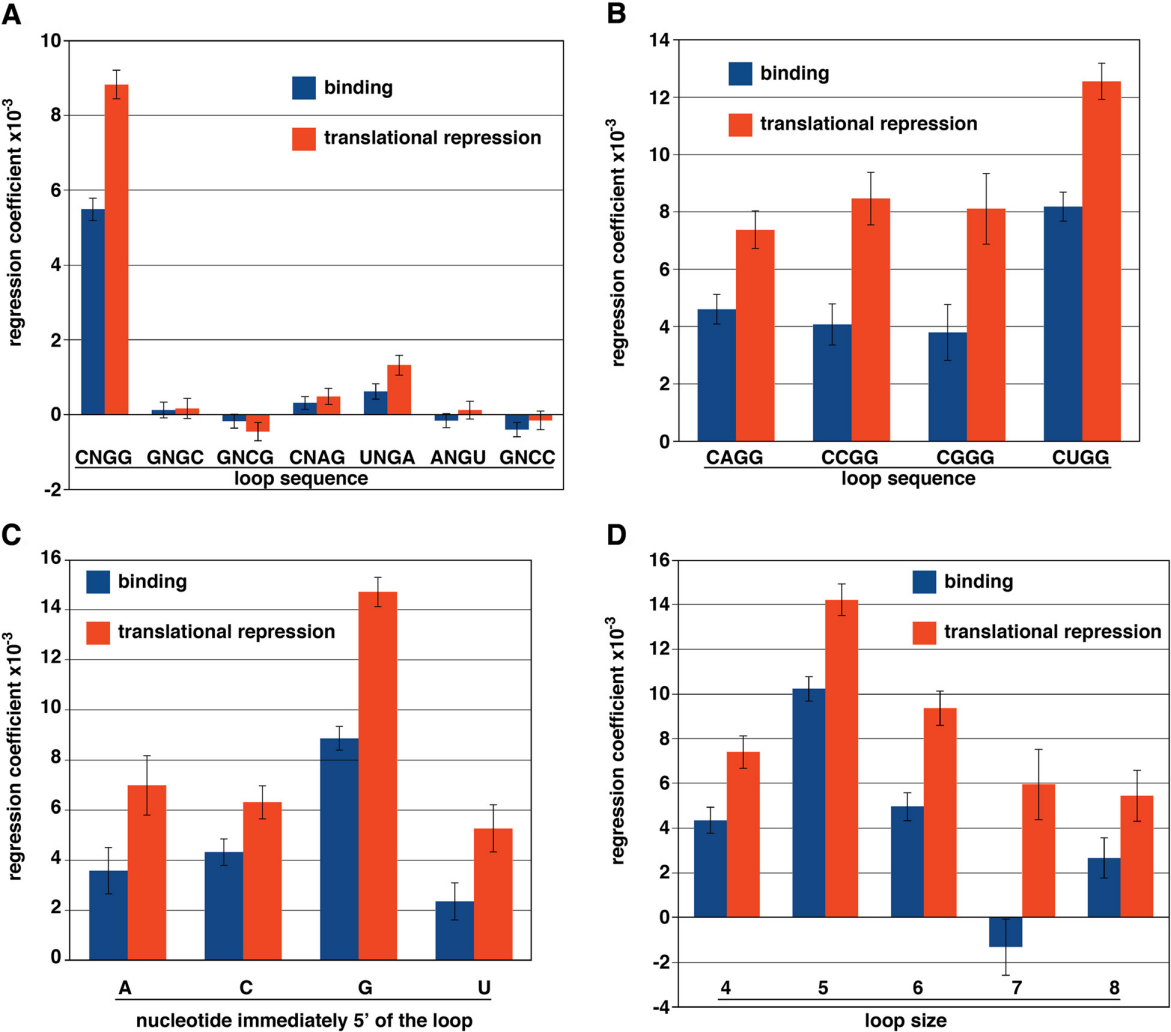
**SREs are enriched in Smaug-bound mRNAs and those that are translationally repressed by Smaug.** Multiple linear regression was used to simultaneously assess the contribution of various stem-loop structures to Smaug binding and Smaug-mediated translational repression. Smaug binding was quantified using the fold enrichment in Smaug RIPs compared to control RIPs and Smaug-mediated translational repression was quantified by comparing the TI in *smaug*-mutant versus wild-type embryos. The structures tested in **(A)** included a consensus SRE with the loop sequence CNGGN_0-4_ while the other sequences tested carried the indicated changes in invariant positions of the loop that are predicted to reduce or block Smaug binding. The structures tested in **(B)** included all possible nucleotides in the second position of the loop. The structures tested in **(C)** included all possible nucleotides in the position that immediately precedes the loop. The structures tested in **(D)** included loops matching the CNGGN_0-4_ consensus where the loop size varied from four to eight nucleotides. Error bars indicate standard error of the mean.

Next, again using fold-enrichment and change in TI as metrics for binding and translational repression, respectively, we employed multiple linear regression to simultaneously assess the possible contributions of stem-loops carrying CNGGN_0-4_ loops along with six altered stem-loops. The altered structures contained changes in the invariant nucleotides in the CNGGN_0-4_ loop that are predicted to lower their affinity for the Smaug RNA-binding domain. We found that the *bona fide* SRE was a significantly better predictor of both Smaug binding and Smaug-mediated translational repression than any of the altered stem-loops (Figure 6A). These results are consistent with positive correlations between the presence of sequences matching the SRE consensus within mRNAs that are translationally repressed and/or degraded in wild-type *Drosophila* embryos [44].

We next used these data sets to explore the predictive power of other SRE features using the same approach. We first tested SRE variants carrying different nucleotides in the N2 position of the loop and found that CUGG performed better than CGGG, CAGG and CCGG loops, the latter three of which were similarly predictive of both Smaug binding and translational repression (Figure 6B). These data are largely consistent with work suggesting that the yeast and human Smaug homologs have binding preferences for SREs bearing CUGG and CGGG loops over CAGG and CCGG [43,45]. We next tested the preference for the nucleotide immediately 5′ to the loop and found that, while A, C and U performed similarly, G performed better (Figure 6C). This result is consistent with the binding specificity determined for the yeast and human Smaug homologs [45-48]. Finally, we tested the effect of varying the SRE loop size and found that loops of five nucleotides performed best of all, with a gradual decrease in the predictive value of shorter or longer loops (Figure 6D).

### Smaug co-regulates translational repression and degradation of a large fraction of its target mRNAs

**Figure 7:**
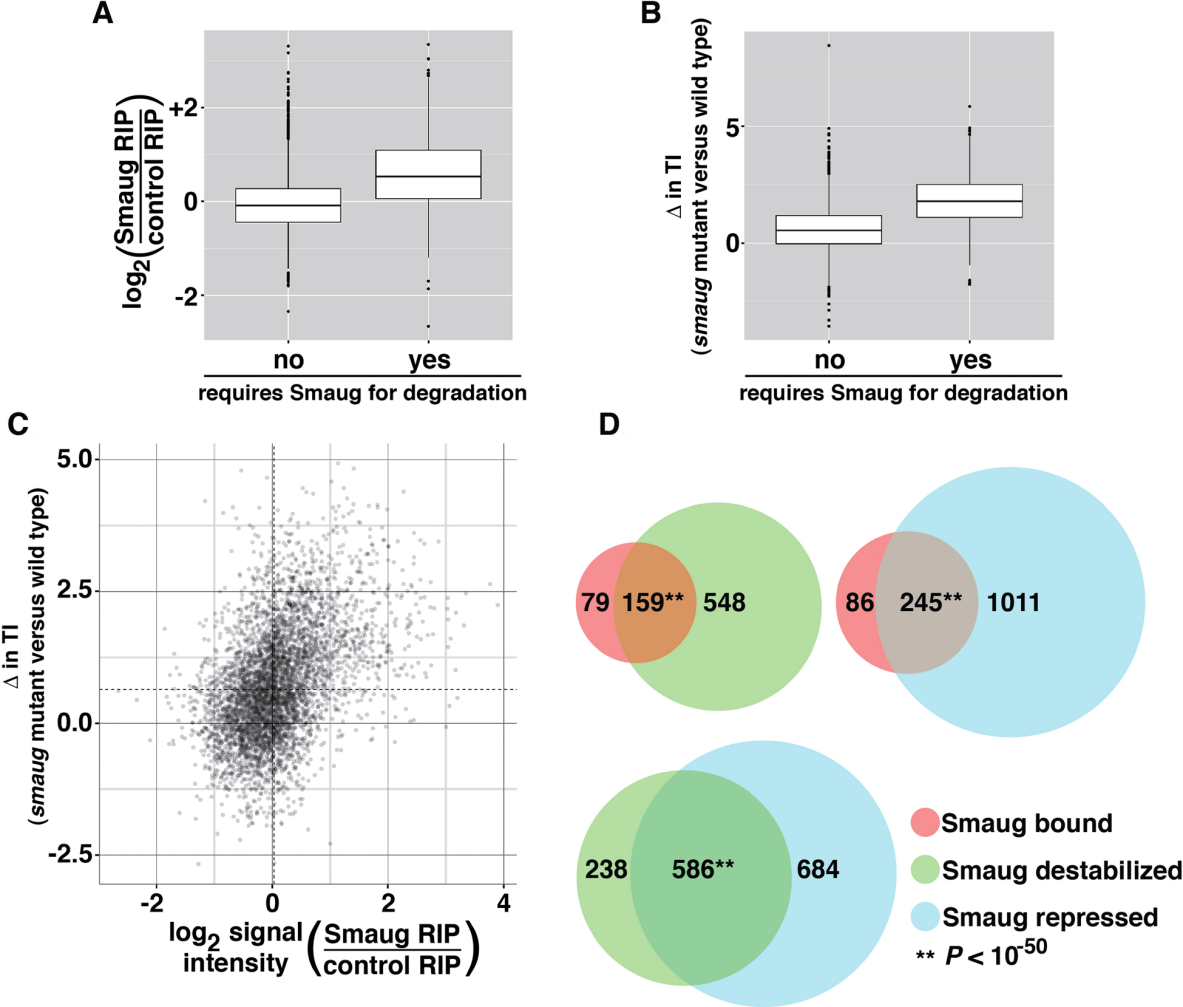
**Comparisons of Smaug-bound, repressed and degraded data sets. (A)** Smaug binding was assessed using the fold enrichment in Smaug RIPs compared to control RIPs and box plots were used to show the range of these enrichments for the targets of Smaug-mediated mRNA decay and for non-targets. (**B)** Smaug-mediated translational repression was assessed using the change in TI in *smaug*-mutant compared to wild type and box plots were used to show the range of these changes for the targets of Smaug-mediated mRNA decay and for non-targets. **(C)** Smaug binding and translational repression were quantified as described in **(A)** and **(B)**, respectively, and these values were plotted against one another. The dashed vertical and horizontal lines represent the median values for Smaug binding and Smaug-mediated translational repression, respectively. **(D)** Venn diagrams to show the overlap between the genes whose mRNAs are bound by Smaug, those that are degraded by Smaug and those that are translationally repressed by Smaug (FDR <10%). Note that, for each comparison, only genes scored as ‘expressed’ in both data sets were included.

Smaug employs different mechanisms to regulate the expression of its two characterized target mRNAs, *nanos* and *Hsp83*[14-16,28,31,33]. To gain a panoramic view of how Smaug regulates its target transcripts we compared the data for Smaug binding and translational repression from the current study to the data from our previous, genome-wide analyses of Smaug-induced transcript decay [9]. For the first set of comparisons the fold-enrichment of an mRNA in Smaug RIPs versus control RIPs was used as a metric for Smaug binding and the change in TI between the *smaug*-mutant and wild type was used as a metric for translational regulation. We found that mRNAs requiring Smaug for their degradation showed significantly higher levels of both Smaug binding (Figure 7A; Wilcoxon rank-sum test, *P* < 3 × 10^-16^) and Smaug-mediated translational repression (Figure 7B; Wilcoxon rank-sum test, *P* < 3 × 10^-16^) than mRNAs whose decay is not regulated by Smaug. Using these two measures we also found a genome-wide correlation between Smaug binding and Smaug-mediated translational repression (Spearman’s rho = 0.43, Fisher’s exact test *P* < 3 × 10^-16^; Figure 7C).

We then compared the lists of genes whose mRNAs are bound by Smaug to those that are degraded or translationally repressed by Smaug (Figure 7D). As described above, our data suggest that several thousand mRNAs are translationally repressed by Smaug and that the calculated FDR overestimates the true FDR [49]. Thus, for all comparisons involving polysome data we used a list of genes whose mRNAs show an increase in TI in *smaug*-mutant embryos versus wild type at an FDR <10% rather than at <5%. This cutoff, often used in place of 5%, is near an inflection point in the plot of gene number versus FDR (Additional file 10), indicating that there is a much higher, and fairly consistent, enrichment for true positives up until that point.

We found that at least 67% of the mRNAs bound by Smaug are targets of Smaug-mediated decay, while at least 74% of the mRNAs bound by Smaug are translationally repressed by Smaug (Figure 7D). We also found a substantial and significant overlap between the lists of genes that encode mRNAs that are translationally repressed by Smaug and those that require Smaug for their degradation (that is, 71% of the mRNAs that are degraded by Smaug are also translationally repressed by Smaug while 46% of mRNAs that are translationally repressed by Smaug are targets of Smaug-mediated mRNA decay; Figure 7D). A comparison of all three data sets can be viewed in Additional file 11. Taken together, these data indicate that a large fraction of Smaug’s targets are both translationally repressed and degraded by Smaug.

**Figure 8:**
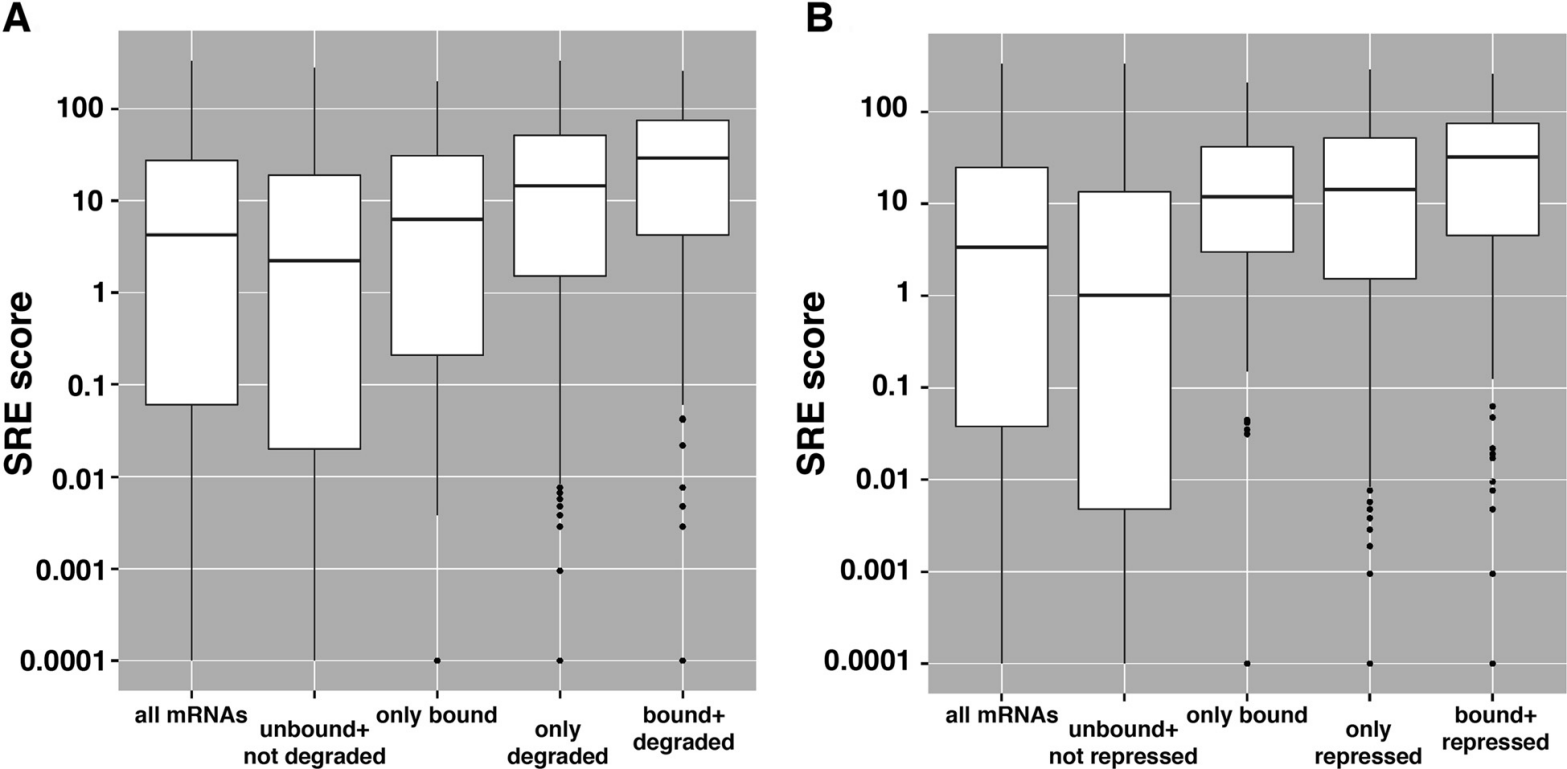
**Smaug-degraded and Smaug-repressed mRNAs are enriched for SREs. (A)** Genes were divided into one of four classes (see the main text for more details): 1) bound and degraded by Smaug; 2) neither bound nor degraded by Smaug; 3) only bound by Smaug; and 4) only degraded by Smaug. The range of SRE scores for these classes is shown in the box plots. **(B)** Genes were divided into one of four classes (see the main text for more details): 1) bound and translationally repressed by Smaug; 2) neither bound nor translationally repressed by Smaug; 3) only bound by Smaug; and 4) only translationally repressed by Smaug. The range of SRE scores for these classes is shown in the box plots. ‘All genes’ shows the range of SREs scores for all of the genes represented in **(A)** and **(B)**, respectively.

The comparisons from Figure 7D identified a substantial number of genes that require Smaug for their degradation or translational repression but do not appear to be bound by Smaug. These transcripts may require Smaug indirectly for their regulation or they may represent false negatives from the RIP-Chip experiments. To assess the latter possibility, we grouped mRNAs into four different classes where Smaug binders were defined as having an FDR in RIP-Chip of <5% and the targets of Smaug-mediated decay were based on the results of Tadros *et al*. [9]. The four classes were: 1) those mRNAs that were bound by Smaug and required Smaug for their degradation (‘bound + degraded’; Figure 8A); 2) those that were neither bound nor degraded by Smaug (‘unbound + not degraded’); 3) those that were bound by Smaug but did not require Smaug for their degradation (‘only bound’); and 4) those that were not bound by Smaug but did require Smaug for their degradation (‘only degraded’). We then assessed the SRE scores for the mRNAs in each of these groups and found a substantially higher SRE enrichment for the mRNAs in the ‘only degraded’ class compared to the ‘unbound + not degraded’ class (Figure 8A; Wilcoxon rank-sum test, *P* < 3 × 10^-16^). Similar results were obtained for Smaug-mediated translational repression (that is, a significantly higher SRE enrichment for the ‘only repressed’ class of mRNAs compared to the ‘unbound + not repressed’ class of mRNAs (Figure 8B; Wilcoxon rank-sum test, *P* < 3 × 10^-16^). Together these data suggest that a large fraction of the mRNAs that require Smaug for their degradation and/or translational repression that were scored as unbound in the RIP-Chip experiments are nonetheless directly bound by Smaug.

The *nanos* mRNA’s SREs are found in the 3′ UTR [14-16] and the *Hsp83* mRNA’s SREs are found in the open reading frame [28,31], raising the possibility that the differential regulation of these transcripts relates to SRE position. To assess this possibility we compared the SRE scores for the 5′ UTR, open reading frame and 3′ UTR of genes that encode mRNAs that are translationally repressed but not degraded by Smaug, degraded by Smaug but not translationally repressed, and both repressed and degraded by Smaug (Additional file 12). These results indicated that the vast majority of SREs are localized within target transcripts’ open reading frames and that SRE location within target mRNAs does not explain their differential regulation by Smaug.

### Subcellular localization of Smaug’s target mRNAs

Given Smaug’s role in controlling the subcellular distribution and expression of localized mRNAs, we analyzed the list of Smaug-bound mRNAs for subcellular localization patterns reported by the Fly-FISH database [6,50]. We searched for enrichment of the Fly-FISH database categories defined in embryonic stages 1 to 3 and 4 to 5, representing the stages from which the Smaug-regulated mRNAs were identified (Additional files 13 and 14). The Fly-FISH database not only categorizes subcellular localization patterns but also reports whether an mRNA is degraded. Consistent with Smaug’s role in transcript degradation, Smaug-bound mRNAs were enriched for the Fly-FISH category ‘degraded’. Additional highly enriched categories were those that describe mRNAs that are localized to the posterior of the embryo (for example, ‘posterior localization’, ‘pole cell enrichment’ and ‘pole cell localization’). Together the Smaug-bound mRNAs that fell into these categories produced a collection of 44 genes, including *nanos* and *Hsp83*, whose mRNAs are localized to the posterior. Of these 44 genes, 38 are regulated by Smaug at the level of mRNA stability and/or translation (Additional file 15).

### Functional analysis of Smaug-regulated mRNAs

To gain insights into Smaug’s biological functions in early embryos we searched the list of Smaug-bound mRNAs for encoded proteins with functions related to known aspects of the *smaug*-mutant phenotype. Embryos that lack Smaug show defects in the cell cycle that are associated with a failure in DNA replication checkpoint activation [11,15], suggesting that Smaug might regulate the expression of genes involved in these processes. Thus, we searched the list of Smaug-bound mRNAs for genes that are annotated to play roles in the cell cycle, checkpoint response and/or response to DNA damage. We found a total of 32 such genes and enrichment for the Gene Ontology (GO) term ‘cellular response to DNA damage’. This list of genes included *cdc2c*, *mitotic 15* (*mit(1)15*), *Replication Protein A 70* (*RpA-70*), *Regulator of cyclin A1* (*Rca1*), *Cyclin E* (*CycE*), *Minichromosome maintenance 3* (*Mcm3*), *CDC45L*, *mutagen-sensitive 201* (*mus201*) and *Msh6*. Of these 32 genes, 29 are regulated by Smaug at the level of mRNA stability and/or translation (Additional file 16).

Smaug also plays a prominent role in activating the transcription of the zygotic genome in the early embryo [11]. We thus searched the list of Smaug-bound mRNAs for genes that are annotated to have roles in transcription and/or chromatin and found a total of 25 genes, including *dre4*, *Polycomblike* (*Pcl*), *Nucleosome assembly protein 1* (*Nap1*), *Nucleosome remodeling factor - 38kD* (*Nurf-38*), *anti-silencing factor 1* (*asf1*), *Caf1-180*, *Caf1-105*, and *vig2*. Of these 25 genes, 24 are regulated by Smaug at the level of mRNA stability and/or translation (Additional file 17).

We also searched for novel functions of Smaug by analyzing the Smaug-bound mRNAs via gene set annotation enrichment analysis using the DAVID annotation tool [51,52] applying two stringencies to the analysis: the standard DAVID FDR cutoff of <10% and the more stringent Benjamini-Hochberg FDR (*P*-value of <0.1). These analyses suggest several previously unrecognized roles for Smaug in the early embryo (Table 1).

**Table 1:** Gene set annotation enrichment analysis results for Smaug-bound mRNAs

**Enriched term or feature**^**a**^	**Enriched with FDR ≤ 10**%^**b**^	**Enriched with ‘Benjamini-Hochberg’ FDR ( *P *-value of <0.1)**^**b**^
Cellular response to DNA damage	√	-
Chaperonin Cpn60/TCP-1 family	√	-
Proteasome regulatory particle/ubiquitin proteasome pathway	√	√
Lipid droplet	√	√
Oxidoreductase	√	√
Glycolysis/gluconeogenesis	√	-

^a^For simplicity, terms with similar meanings are clustered or represented with a single GO term, SwissProt keyword, or UniProt feature.

^b^Enrichment analysis was performed using the DAVID functional annotation tool.

First, Smaug may play a role in regulation of protein folding. For example, Smaug-bound mRNAs encode five proteins (Hsp60, T-cp1ζ, CG5525, CG8258 and CG7033) that are members of the Chaperonin Cpn60/TCP-1 family as defined by the Interpro database and are involved in protein folding. The last four of these proteins are subunits of the eukaryotic chaperonin TCP1-ring complex (TRiC), also known as the chaperonin containing TCP-1 (CCT), which consists of two rings composed of eight different subunits [53]. Consistent with a role for Smaug in regulating protein folding, all five of these genes are regulated by Smaug at the level of translation and/or mRNA stability (Additional file 18).

Second, Smaug-associated mRNAs are enriched for the related GO terms ‘proteasome regulatory particle’ and ‘proteasome complex’ as well as the Protein Analysis Through Evolutionary Relationships (PANTHER) term ‘ubiquitin proteasome pathway’. The ubiquitin proteasome system plays a vital part in a variety of cellular processes through its role in the degradation of target proteins. This mechanism involves the post-translational addition of multiple ubiquitin moieties onto a protein, which, in turn, target the protein for proteasomal degradation [54]. The 26S proteasome consists of a 20S core particle, which carries the proteasome’s proteolytic activity, and either one or two 19S regulatory particles, which are necessary for proteasome activity and are composed of 19 subunits [54]. Strikingly, Smaug associates with nine of the mRNAs that encode the regulatory subunits (*Regulatory particle triple-A ATPase 3* (*Rpt3*), *Regulatory particle triple-A ATPase 5* (*Rpt5*), *Regulatory particle non-ATPase 1* (*Rpn1*), *Regulatory particle non-ATPase 2* (*Rpn2*), *Regulatory particle non-ATPase 7* (*Rpn7*), *Regulatory particle non-ATPase 9* (*Rpn9*), *Regulatory particle non-ATPase 10* (*Rpn10*), *Regulatory particle non-ATPase 11* (*Rpn11*) and *Regulatory particle non-ATPase 13* (*Rpn13*)). In contrast, Smaug does not interact with any of the mRNAs that encode the 20S core particle proteins. In addition, Smaug interacts with mRNAs that encode proteins involved in other aspects of the ubiquitin-proteasome system (*Ubiquitin activating enzyme 1* (*Uba1*), *Ubiquitin fusion-degradation 1-like* (*Ufd1-like*), *TER94* and *CG9588*). Consistent with a role for Smaug in control of the ubiquitin-proteasome system, 12 out of these 13 mRNAs (Additional file 19), including all of the transcripts that encode regulatory subunit proteins, are regulated by Smaug at the level of translation and/or mRNA stability.

Third, Smaug might play a role in regulating lipid storage and/or mobilization since the GO term ‘lipid droplet’ is enriched in the Smaug-bound mRNAs. Lipid droplets are ubiquitous organelles that are found in a wide range of organisms from bacteria to humans. They consist of a neutral-lipid core composed of triacylglycerols and sterol esters surrounded by a phospholipid monolayer, and they serve as storage sites for energy, sterols and membrane precursors [55]. Several studies have used proteomic approaches to identify lipid droplet-associated proteins, including two studies that purified lipid droplets from *Drosophila* fat-body tissue or from *Drosophila* embryos [56,57]. Comparison of those lists with our data identified 33 Smaug-bound mRNAs that encode lipid droplet-associated proteins. In addition, our data indicated that 29 of these 33 mRNAs are destabilized and/or translationally repressed by Smaug (Additional file 20). Taken together these data suggest that Smaug may control aspects of lipid droplet function through its regulation of these mRNAs.

Fourth, a direct role for Smaug in regulation of metabolism is suggested by the enrichment for terms such as the SwissProt keywords ‘oxidoreductase’ and ‘NAD’ and the GO terms ‘oxidation reduction’ and ‘cofactor binding’ within Smaug-bound mRNAs. Together these lists comprise a total of 37 metabolic enzymes that function in a wide variety of pathways, including fatty acid metabolism, pyruvate metabolism, amino acid metabolism, the citric acid cycle and oxidative phosphorylation. Our data suggested that 28 out of 37 of these genes are regulated by Smaug at the level of mRNA stability and/or translation (Additional file 21). In addition, we found enrichment for the GO term ‘glucose metabolic process’ and the Kyoto Encyclopedia of Genes and Genomes (KEGG) pathway ‘glycolysis/gluconeogenesis’. These lists contain nine genes, including four encoding enzymes of the glycolytic pathway (*Hexokinase A* (*Hex-A*), *Phosphoglycerate kinase* (*Pgk*), *Phosphoglucose isomerase* (*Pgi*) and both genes encoding *Glyceraldehyde 3 phosphate dehydrogenase* (*GAPDH1* and *GAPDH2*)) and our data indicated that all nine are regulated by Smaug at the level of stability and/or translation repression (Additional file 22). Furthermore, our data suggest that mRNAs encoding four additional glycolytic enzymes may be regulated by Smaug. *Phosphofructokinase* (*Pfk*) and *Triose phosphate isomerase* (*Tpi*) have FDRs in the RIP-Chip data of 5.15% and 6.08%, respectively, and both are targets of Smaug-mediated transcript degradation and translational repression (Additional file 22). Also, *Enolase* (*Eno*) and *Pyruvate kinase* (*Pyk*) are regulated by Smaug at the level of stability and/or translation. In summary, our data suggest that 8 of the 10 glycolytic enzymes may be regulated by Smaug.

### Validation of Smaug’s role in regulation of target mRNAs

To assess the role of Smaug in regulating the expression of the new target mRNAs, we selected five for further analysis: *Rpn7*, *Hexokinase*, *Phosphofructokinase*, *Su(z)12*, and *Bicaudal C*. Rpn7 is a proteasome regulatory particle subunit and was selected because of the observed enrichment for GO terms related to ‘proteasome regulatory particle’. Likewise, because of enrichment for the GO term ‘glucose metabolic process’ and the KEGG pathway ‘glycolysis/gluconeogenesis’, we assayed hexokinase, the first enzyme in glycolysis, and phosphofructokinase, which represents a critical point of regulation [58,59] and catalyzes the committed step of glycolysis (that is, the product of this reaction serves solely as a precursor to the final product of the glycolytic pathway). Polycomb repressive complex 2 (PRC2) trimethylates histone H3 on lysine 27, a mark that is associated with transcriptional silencing [60]. Thus, Su(z)12, a component of PRC2, was of interest in light of the failure to induce zygotic transcription in *smaug*-mutant embryos [11]. Bicaudal C is an RNA-binding protein that represses the translation of target mRNAs during *Drosophila* oogenesis [61]. Thus, Bicaudal C overexpression in *smaug*-mutant embryos could disrupt normal patterns of post-transcriptional regulation.

**Figure 9:**
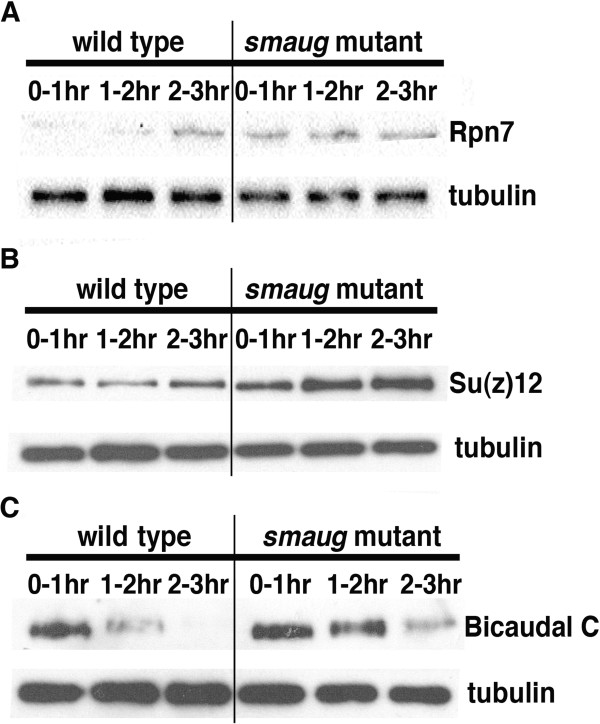
**Validation of new Smaug targets.** Extracts were prepared from 0- to 1-, 1- to 2- and 2- to 3-hour-old wild-type and *smaug*-mutant embryos and assayed for the levels of **(A)** Rpn7, **(B)** Su(z)12 and **(C)** Bicaudal C proteins via western blots.

**Figure 10:**
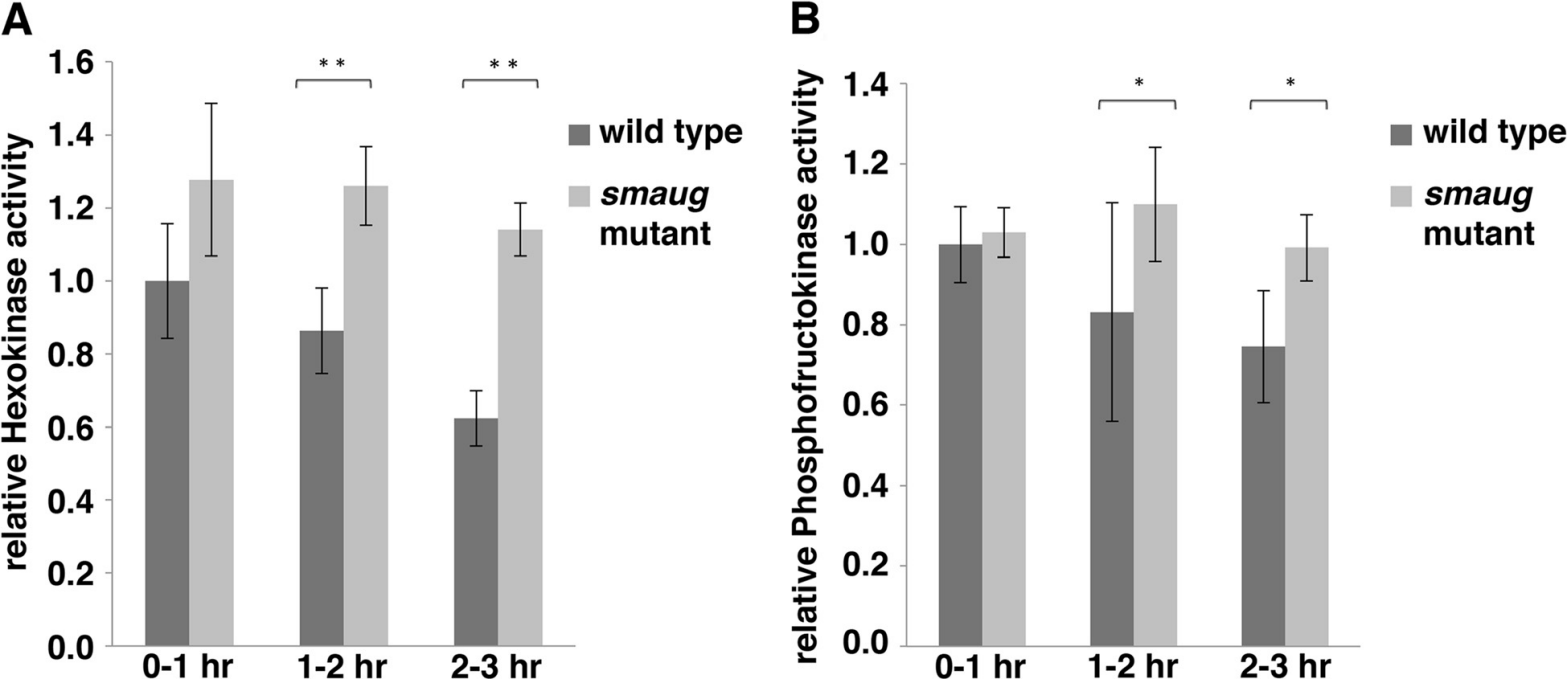
**Glycolytic enzymes are overexpressed in *smaug*-mutant embryos.** Extracts were prepared from 0- to 1-, 1- to 2- and 2- to 3-hour old wild-type and *smaug*-mutant embryos and assayed for **(A)** hexokinase activity or **(B)** phosphofructokinase activity. Activities are shown relative to the wild type 0 to 1 hour time point in each case. Results are the average of three independent experiments and error bars indicate standard error of the mean. Data were analyzed using a Student’s *t*-test (**P < 0.05, *0.05 < *P* <0.1).

Western blots (Rpn7, Su(z)12, Bicaudal C; Figure 9) or enzyme activity assays (hexokinase, phosphofructokinase; Figure 10) showed that, in all cases, there was an increase in expression in *smaug*-mutant embryos versus wild-type ones (Figures 9 and 10), consistent with a role for Smaug in down-regulation of its new target transcripts.

## Discussion

Here we have used genome-wide approaches to identify mRNAs that are bound by Smaug and those that are translationally repressed by Smaug. Our results show that the presence of SREs is predictive of both binding and translational repression and, consistent with previous work on the yeast and human Smaug homologs [43,45-48], indicate that the *Drosophila* SRE consensus is more restricted than previously thought [17]. Integration of these new results with our earlier ones on Smaug’s global role in mRNA decay [9] has led to the following conclusions: 1) Smaug directly regulates the expression of a large number of mRNAs; 2) a large fraction of Smaug-regulated transcripts are both destabilized and translationally repressed; and 3) Smaug plays a key role in controlling the expression of mRNAs localized to the posterior of the embryo. In addition, we have uncovered new and unanticipated roles for Smaug in regulation of protein folding and decay, as well as in metabolism.

### Translational repression versus mRNA decay

Previous work has firmly established that Smaug can both repress translation and induce degradation of target mRNAs. However, Smaug’s two well-characterized target transcripts, *nanos* and *Hsp83*, are differentially regulated by Smaug [14-16,28,31,33]. The work presented here suggests that, unlike *nanos* and *Hsp83*, Smaug both translationally represses and degrades a large fraction of its target mRNAs. We hypothesize that the extent to which Smaug regulates the translational repression and/or destabilization of its targets is likely to be a consequence of additional *cis*-elements within target mRNAs. For example, the *Hsp83* 3′ UTR contains a translational enhancer that may mitigate Smaug-mediated translational repression [62]. Similarly, the modest stabilization of *nanos* mRNA observed in the absence of Smaug suggests that additional *cis*-elements within the *nanos* transcript function in its destabilization.

### Smaug’s role in the regulation of posterior-localized mRNAs

Smaug functions in the localization and regulation of its target mRNAs at the posterior of the embryo [14-16,28,31,34-37]. This is a consequence of Smaug’s ability to induce transcript decay and to repress translation in the bulk cytoplasm of the embryo combined with mechanisms that inactivate Smaug function in the germ plasm at the posterior. Indeed, we have found that 38 of the 44 posterior-localized mRNAs that are bound to Smaug are regulated by Smaug at the level of stability and/or translation.

A critical aspect of Smaug’s role in the regulation of *nanos* and *Hsp83* mRNA is the fact that transcripts found at the posterior of the embryo escape Smaug regulation. The molecular mechanisms that underlie this spatial regulation of Smaug function are not understood, but Oskar protein has been implicated in blocking Smaug function at the posterior and has been shown to physically interact with Smaug [14,15,36,63]. Indeed, it has been shown that Oskar’s interaction with Smaug blocks Smaug’s ability to bind to its target mRNAs and it has therefore been proposed that the Oskar-Smaug interaction blocks Smaug function by preventing Smaug’s interaction with its target transcripts [30,64]. This simple model, however, is not consistent with work showing that a *torso* mRNA carrying the first 96 nucleotides of the *nanos* mRNA’s 3′ UTR, which includes one of the *nanos* SREs, is repressed at both the anterior and posterior of the embryo [14]. In addition, a *torso* mRNA carrying the first 185 nucleotides of the *nanos* 3′ UTR, which contains both *nanos* SREs, is repressed at the anterior but is expressed at the posterior [65]. Taken together these data suggest the existence of one or more *cis*-elements mapping within nucleotides 97 to 185 of the *nanos* 3′ UTR that localize *nanos* transcripts to the germ plasm [66] and/or abrogate Smaug’s ability to repress *nanos* mRNA expression in the germ plasm. Our identification of several dozen posterior-localized, Smaug-bound transcripts should facilitate identification of any additional *cis*-elements.

### Identification of new biological functions for Smaug

Our analysis of the mRNAs that are bound by Smaug has identified a number of mRNAs that encode proteins that are involved in cell-cycle control and transcriptional regulation. Mis-regulation of one or more of these mRNAs could underlie the cell-cycle and transcriptional defects that occur in the absence of Smaug. Our data also suggest that Smaug has several new and unanticipated biological functions, including control of protein folding and degradation, lipid droplet function and basic metabolism.

#### Protein folding and stability

Our data suggest that Smaug downregulates the expression of 9 of the 19 subunits of the proteasome regulatory particle and 4 out of the 8 that encode the TRiC/CCT complex. In addition, three of the four remaining TRiC/CCT mRNAs and eight of the remaining ten proteasome regulatory particle mRNAs require Smaug for their degradation and/or translational repression (Additional files 18 and 19). It is unclear at this time whether these additional mRNAs represent false negatives in the RIP-Chip experiments or whether Smaug regulates their expression indirectly. Nonetheless, our data indicate that Smaug regulates the expression of almost all of the components of these two protein complexes. Previous work has shown that proteasome levels are repressed in early embryos [67] and our data suggest that Smaug plays a major role in this repression. Given the role of the proteasome in cell-cycle regulation [68], Smaug-mediated regulation of the proteasome may underlie some or all of the cell-cycle defects observed in *smaug* mutants.

#### Lipid droplets

Previous experiments to characterize lipid droplet-associated proteins in embryos employed six independent purifications and grouped the identified proteins based on the number of purifications in which they were detected [57]. They found 127 that were identified in at least three purifications and 453 that were identified in one or two runs. Of the 28 Smaug-bound mRNAs that encode lipid-droplet proteins, 22 were identified in three or more runs, suggesting that Smaug regulates mRNAs that encode proteins abundant in and/or tightly associated with lipid droplets.

Lipid droplets are storage sites of triacylglycerols, hydrolysis of which yields fatty acids that can be metabolized for energy or serve as a source of membrane precursors. Thus, lipid droplets could function as the source of membrane precursors that are required during blastoderm cellularization, a process during which plasma membrane invaginates around the syncytial nuclei that are found at the embryo’s periphery. A role for Smaug in regulating lipid droplet function is intriguing as *smaug* mutant embryos show defects in cellularization. In addition, given the possible use of fatty acids as an energy source, Smaug’s regulation of lipid droplet function could also reflect Smaug’s more general role in control of metabolic processes (see below).

#### Metabolism

Our data also suggest a widespread role for Smaug in regulating metabolism in the early embryo, including a role for Smaug in down-regulation of glycolysis. Previous work has suggested that maternal mRNAs encoding the glycolytic enzymes are present in early *Drosophila* embryos but are rapidly degraded [69-75]. Glycolysis is down-regulated, not only in *Drosophila*, but also in frog and mammalian early embryos [76,77] but the molecular mechanisms involved are unknown. Our data implicate Smaug in the degradation and/or translational repression of many of the glycolytic mRNAs. It will be interesting to test whether post-transcriptional regulation of these mRNAs by Smaug’s homologs plays a role in the early embryos of all animals.

### Biological implications of the large number of Smaug-target mRNAs

Our data are consistent with Smaug directly regulating a large number of mRNAs in early embryos through translational repression and/or transcript degradation. This raises the question as to whether all of these repressive interactions are biologically important.

In one model only a subset of Smaug’s targets are biologically relevant because the extent of downregulation by Smaug varies in a target-dependent manner. For the biologically relevant target transcripts, Smaug would effectively turn off their expression while, for the others, Smaug would reduce their expression insufficiently to have an effect on their biological function. A similar type of model has been suggested for repression mediated by individual miRNAs, which, as in the case of Smaug, regulate the expression of a large number of transcripts [78]. Given the low complexity of the binding sites of most RNA-binding proteins it is likely that many of the *trans*-acting factors that control mRNA translation and/or stability will regulate a large number of transcripts and, as such, the same concepts should apply.

An alternative, but not mutually exclusive, model is that factors like Smaug, which repress the expression of a large number of mRNAs, do so in order to limit the total levels of available mRNA within a cell. This reduction could result from both Smaug-directed degradation of transcripts and/or Smaug-mediated translational repression, the former eliminating the mRNAs and the latter removing them from the pool of available mRNAs. In this model, Smaug would function to control the competition among transcripts for limiting cellular components, such as the translation machinery. We note, however, that our data do not support this model - at least in regard to the translation machinery - as we fail to see a decrease in the translation of mRNAs that are not bound by Smaug in *smaug*-mutant embryos.

A third model to explain the biological significance of the regulation of a large number of mRNAs by a single factor relates to a requirement for large-scale changes in a cell’s function. Under such a circumstance one might expect that the expression of a large number of mRNAs must be translationally repressed and/or degraded while a new group of genes is activated. For example, during the first 2 to 3 hours of *Drosophila* embryogenesis, nuclei are transcriptionally silent and development is driven by mRNAs synthesized by the mother and deposited into the egg during oogenesis. Subsequently, one- to two-thirds of these maternal mRNAs are degraded [4,9,12] - the majority in a Smaug-dependent manner - concurrent with activation of transcription in embryonic nuclei. In the early embryo this widespread degradation appears to serve at least two purposes. The first involves clearing the embryo of mRNAs that are no longer required. In the second, ubiquitously distributed mRNAs are degraded but locally protected from decay [28,37] or are degraded everywhere and then subsequently re-expressed in spatially restricted patterns through transcriptional activation in select embryonic nuclei [4]. Thus, Smaug, through its regulation of a large number of mRNAs, may play a major role in producing spatial precision in gene expression during the maternal-to-zygotic transition in early embryos.

## Conclusions

Smaug directly regulates the expression of a large number of mRNAs in the early *Drosophila* embryo and a significant fraction of these mRNAs are both translationally repressed and destabilized. Smaug plays a major role in controlling the expression of mRNAs that are localized to the posterior of the embryo and regulates a diverse set of processes, including metabolism, lipid droplet function, protein folding and protein stability.

## Materials and methods

### *Drosophila* stocks

Wild-type flies consisted of the *w*^*1118*^ stock maintained in a large-scale *Drosophila* culture. *smaug* mutant alleles included *smaug*^*1*^[15] and *smaug*^*47*^. The *smaug*^*47*^ allele was generated via imprecise excision of a P-element (*GE21229*) using standard methods [79]. *GE21229* is inserted 2,499 bp 5′ of the *smaug* start codon and 20 bp downsteam of the transcriptional start site of the *smaug-RB* isoform. All isoforms are defined as described at [80]. The original *smaug*^*1*^ allele showed homozygous maternal effect lethality [15] and we recovered six excision lines demonstrating this phenotype. The extent of the deletion in these six lines was determined via PCR analysis of genomic DNA. Two of the lines, *smaug*^*30*^ and *smaug*^*47*^, showed deletions removing large portions of the *smaug* gene, but not affecting the neighboring upstream and downstream genes - *CG5087* and *CG5280*, respectively. Sequencing revealed that the *smaug*^*30*^ allele is a 4,514 bp deletion of the *smaug* gene beginning 2,480 bp 5′ of and ending 2,034 bp 3′ of the *smaug* start codon. Sequencing also showed that this allele retains 933 bp of the P-element. This deletion removes 2,020 of 2,997 bp of the open reading frame of *smaug RA*, *RB*, *RC*, and *RE* isoforms. The *smaug*^*47*^ allele is a 5,542 bp deletion beginning 2,483 bp 5′ of and ending 3,059 bp 3′ of the *smaug* start codon. This deletion leaves 39 bp of the open reading frame in the *smaug RA*, *RB*, *RC*, and *RE* isoforms.

### RNA co-immunoprecipitations

Embryos collected at 0 to 3 hours post-egglaying were dechorionated with 50% bleach and homogenized in a minimal volume of RIP lysis buffer (150 mM KCl, 20 mM HEPES pH 7.4, 1 mM MgCl_2_, 1 mM dithiothreitol (DTT), 1× protease inhibitor cocktail (Bioshop, Burlington, Ontario, Canada)). Extracts were centrifuged for 10 minutes at 4°C, and the supernatant was supplemented with 9 M urea to a final concentration of 2 M. Protein A beads were pre-incubated with either guinea pig anti-Smaug antibody [9] or normal guinea pig serum followed by four washes with RIP lysis buffer supplemented with urea. These beads were then incubated with embryo extract for 2 h at 4°C followed by four washes with RIP lysis buffer supplemented with urea and RNA was extracted from the beads using the Trizol reagent (Life Technologies, Burlington, Ontario, Canada).

### Polysome gradients

Embryos laid by wild-type or *smaug*^*1*^ homozygous mothers were collected 0 to 2 hours post-egglaying, dechorionated with 100% bleach and lysed in an equal volume of polysome lysis buffer (7.5 mM MgCl_2_, 500 mM NaCl, 25 mM Tris pH 7.5, 2 mg/ml heparin, 0.5 mg/ml cycloheximide, 1 mM DTT, 50 U/ml RNasin, 1 mM 4-(2-aminoethyl) benzenesulfonyl fluoride hydrochloride (AEBSF), 2 μg/ml leupeptin, 2 mM benzamidine, 2 μg/ml pepstatin A). Lysed samples were diluted 1 in 12.5 in polysome lysis buffer and 30% triton was added to a final concentration of 1% and then spun at 6,000xg for 10 minutes and the resulting supernatant was diluted in polysome lysis buffer supplemented with 1% Triton to an A260 of 12.5.

A 12 ml 15% to 45% linear sucrose gradient in 7.5 mM MgCl_2_, 500 mM NaCl, 50 mM Tris pH 7.5 was created using a BioComp Model 117 Gradient Mate gradient maker (Biocomp, Fredericton, New Brunswick, Canada) using a rotation angle of 80.5° and a rotation speed of 18 rpm for 1 minute and 58 seconds. After chilling the polysome gradient on ice, 400 μl of diluted embryo extract was loaded onto the top of the gradient, which was then spun at 36,000 rpm in a Beckman SW 41 Ti rotor for 2.5 hours. The gradients were then separated into four pools (pool 1 contained the top 4 ml, pool 2 contained the next 2 ml, pool 3 contained the next 3 ml and pool 4 contained the last 3 ml and the pellet). A fixed amount of exogenous *in vitro* transcribed *Arabidopsis* spike-in RNAs was then added to each pool. Our microarrays contain probes that allow for the detection of these RNAs allowing for subsequent data normalization. We added 20% SDS, 0.5 M EDTA and 20 mg/ml proteinase K to each fraction to final concentrations of 0.8%, 0.01 M and 0.128 mg/ml, respectively, and then incubated them for 30 minutes at room temperature. Glycogen was then added to a final concentration of 80 μg/ml and samples were ethanol precipitated overnight and the resulting pellet was washed with 75% ethanol and resuspended in phenol-saturated water. Following two phenol-chloroform extractions, samples were precipitated by the addition of 7.5 M LiCl to a final concentration of 1.5 M and an overnight incubation at 4°C. The resulting pellet was washed with 75% ethanol, resuspended in water and ethanol precipitated in the presence of 80 μg/ml of glycogen and 0.3 M sodium acetate. The precipitate was then washed with 75% ethanol and resuspended in water. The integrity of RNA in each pool was confirmed via northern blots, which were probed for *nanos* mRNA (Additional file 23).

Experiments that utilized EDTA treatment involved lysis of embryos in polysome lysis buffer and the resulting sample was split in two and the polysome gradient experiment proceeded as described above with the following changes. One sample was diluted into polysome lysis buffer and fractionated as normal, while the other was diluted in polysome lysis buffer lacking MgCl_2_ and containing 25 mM EDTA and fractionated on gradients containing 25 mM EDTA and lacking MgCl_2_. After centrifugation these gradients were divided into 12 1-ml fractions and RNA was extracted from each fraction and analyzed via northern blot.

For experiments that utilized puromycin embryos were lysed in puromycin lysis buffer (50 mM Tris pH 7.5, 2 mM MgCl_2_, 500 mM KCl, 100 μM GTP, 1 mM DTT, 50 U/ml RNasin, 1 mM AEBSF, 2 μg/ml leupeptin, 2 mM benzamidine, 2 μg/ml pepstatin A). The lysed samples were split in half and cycloheximide was added to one sample to a final concentration of 0.5 mg/ml and puromycin was added to the other sample to a final concentration of 2 mM. Samples were left on ice for 20 minutes and then incubated at 30°C for 10 minutes. Both samples were then diluted 1 in 12.5 with polysome lysis buffer supplemented with either puromycin or cycloheximide and 30% triton was added to a final concentration of 1%. The samples were then spun at 6,000xg for 10 minutes and the supernatant was diluted with polysome lysis buffer supplemented with either puromycin or cycloheximide to give an A260 of 12.5 and these diluted samples were then fractionated as described above.

### Microarrays

RNA samples from RIP experiments were used to prepare single-stranded cDNA using anchored oligo(dT) primers and the Canadian *Drosophila* Microarray Centre indirect labeling protocol, which can be viewed at [81]. Anchored oligo(dT) primers consist of 20 T residues and end in an A, C or G residue followed by an A, C, G or T. Thus, priming occurs only at the 5′ end of the poly(A) tail and transcripts with short tails will be primed with equal efficiency to those that have long tails. RNA samples from polysome experiments were used to generate double-stranded cDNA following the protocol described in the NimbleGen Array User’s Guide (Gene Expression Arrays, version 5.0) using all reagents at half the normal amount and a primer mixture of random hexamer primers and anchored-oligo-dT primers. Cy3 or Cy5-tagged random nonamers were then used to label cDNAs using the Roche NimbleGen (Madison, Wisconsin, USA) protocol. The cDNA resulting from RIP experiments was used to probe Nimblegen 4x72K arrays (Gene Expression Omnibus (GEO) platform number GPL13782), while the cDNA from polysome gradients was used to probe a custom-designed *Drosophila* 4x72K NimbleGen array (GEO platform number GPL10539) that contain probes for *Arabidopsis* spike-in RNAs (see below). Microarrays were scanned using Genepix Pro software on a Molecular Devices (Sunnyvale, California, USA) GenePix 4000B or 4300A scanner and quantified using Nimblescan.

RIP microarrays were normalized using the Robust Multi-array Average (RMA) quantile method and transcripts that were expressed at levels significantly above background in total RNA collected 0 to 3 hours post-egglaying were determined using ‘one class unpaired analysis’ in SAM and transcripts with an FDR >5% were excluded from further analysis of the RIP data. mRNAs that were reproducibly enriched in Smaug RIPs versus control RIPs were then identified by comparing the log_2_(Smaug IP/Total RNA) and the log_2_(Mock IP/Total RNA) using ‘two class unpaired analysis’ in SAM (FDR <5%).

Polysome microarrays were normalized using the RMA quantile method. We further normalized the data using *Arabidopsis* spike-in RNAs. The hybridization signals from the spike-in RNAs were utilized by applying a linear transformation to each sample with the parameters, *a* and *b*, determined by fitting the linear function *Y = aX + b* using the spike-in signal, where *X* is the expression level of the spike-in RNAs in a specific sample, and *Y* is the mean expression level of the spike-in RNAs across all the samples. The genes significantly expressed in wild-type or *smaug*-mutant embryos in each of pools 1, 2, 3 and 4 were separately determined using ‘one class unpaired analysis’ in SAM (FDR <5%). We defined the genes significantly expressed in the wild-type and *smaug*-mutant embryos as the union of the significantly expressed genes from the four fractions derived from that genotype. We then compared these two lists and defined their intersection as the list of genes significantly expressed in both wild-type and *smaug*-mutant embryos, and restricted all the following analysis to the genes on this list. To determine the list of genes with different polysome association in wild-type and *smaug* mutants, we compared the geometric mean of the expression level in pools 3 and 4 (normalized to the levels in pool 1) in wild-type and *smaug*-mutant embryos, using ‘two class unpaired analysis’ in SAM.

### RT-qPCR

cDNA was synthesized using SuperScript II reverse transcriptase (Invitrogen) and random primers according to the manufacturer’s instructions. Quantitative PCR reactions were carried out using the BioRad (Mississauga, Ontario, Canada) Real-time PCR system as per the manufacturer’s instructions*.* Levels of *RpL32* mRNA in each immunoprecipitated sample were used to normalize the levels of the experimental mRNA in that sample.

### Estimating the number of genes that are translationally repressed by Smaug

The fraction of genes expected to have changed in TI in *smaug*-mutant and wild-type embryo samples for the top *N* and bottom *N* Smaug-binders (for N = 250, 500, and 1,000) was calculated using the R (version 2.14.1) algorithm sm.density() in the sm package (version 2.2-4.1). The sm.density() algorithm provided smoothed density estimates for 100 values of change in TI for the top and bottom N binders, with the 100 values calculated by the sm.density() algorithm with each smoothed density estimate.

For every gene expressed in our polysome gradient experiments, the probability that it was a positive target (that is, a target of Smaug-mediated repression) was estimated using the top *N* and bottom *N* Smaug-binders (for N = 250, 500, and 1,000). First, for each gene, the density of its change in TI under the positive and negative distributions as defined by *N* top and bottom binders, respectively, was set to be equal to that of the closest grid point higher than the change in TI. We then estimated the probability that a gene was a positive by taking the ratio of its density under the positive distribution and the sum of its densities under the positive and negative distributions. This procedure was repeated for each of our three sets of positive and negative distributions to give us three different sets of probabilities. For each of these three sets of probabilities, we estimated the expected number of Smaug targets for that set by summing the ‘positive probabilities’ for all genes.

### Smaug recognition element searching

We used a two-step procedure to computationally predict SRE stem/loops carrying the loop sequence CNGGN_0-4_ on a non-specific stem. First, we performed an initial scan using RNAplfold (version 2.0.7) [82] with the parameters set to -W = 170, -L = 120, -T = 25 choosing these parameter values as they were within the range suggested by Lange *et al.*[83]. Potential SREs for further analysis were identified as CNGG sequences where the base immediately 5′ to the CNGG sequence was involved in a canonical base pair with one of five nucleotides immediately 3′ to the CNGG sequence with probability >0.01. We estimated the probability of formation of an actual SRE (that is, CNGG at the 5′ end of the hairpin loop and a loop of length four to eight nucleotides) at each candidate site using the RNAsubopt [84] routine from the Vienna RNA package. In particular, we sampled 3,000 structures for each of a series of windows overlapping the candidate site (from the Boltzmann ensemble using the ‘-p’ option), computed the empirical probability of SRE formation in each window, and set the SRE probability for a site to be the average of these probabilities. The most 5′ of the sequence windows spanned 75 nucleotides upstream of the candidate site, the site itself, and the 40 nucleotides downstream of the site. The most 3′ of the windows spanned 40 nucleotides upstream of the site to 75 nucleotides downstream. Between these two, all of the other windows were offset by a single nucleotide. These site probabilities were then summarized at the transcript level. The initial SRE score for each transcript was the sum of the SRE probability values at each candidate site within the entire transcript. The same procedure was used to search for CNGG sequence variants and calculate a variant score for each transcript. Once obtained, SRE scores and the scores of sequence variants were compared with polysome and RIP data using standard R packages. Spearman’s correlation values across all of the expressed genes were determined using the cor.test() algorithm with default parameters and the Spearman method. Linear models were created using the lm() algorithm with default parameters.

### Localization pattern enrichment analysis

These analyses were carried out as described in Laver *et al.*[85].

### Western blots

Antibodies against Rpn7 (Santa Cruz Biotechnology, Dallas, Texas, USA; catalogue #SC-65750), Su(z)12 [86] and Bicaudal C [87] were used in standard western blot assays.

### Glycolytic enzyme assays

For enzyme assays *smaug*-mutant embryos were collected from females homozygous for the *smaug*^*47*^ allele, while 'wild-type' embryos were collected from females homozygous for the *smaug*^*47*^ allele that were also homozygous for a genomic *smaug* rescue transgene that was inserted at the attP40 site on the second chromosome by Genetic Services (Cambridge, Massachusetts, USA) using PhiC31 integrase-mediated transgenesis [88]. The *smaug* transgene, which rescues the *smaug* mutant phenotype, is a modified version of a previously generated *smaug* rescue construct [15] that expresses a version of Smaug that is tagged at its amino terminus with FLAG and p53 epitope tags.

For the hexokinase assay, embryos were homogenized in extraction buffer (0.05 M Tris–HCl, pH 8.0 with 13.3 mM MgCl_2_) and assayed in extraction buffer supplemented with 16.5 mM ATP, 20 mM beta-NADP and 0.67 M glucose. Hexokinase catalytic activity was measured by adding *Leuconostoc mesenteroides* glucose-6-phosphate dehydrogenase (Sigma-Aldrich Chemicals, Oakville, Ontario, Canada; Worthington code ZF or ZFL) dissolved at a concentration of 300 IU/ml in extraction buffer. The production of beta-NADPH was monitored at 340 nm in a Thermo SPECTRONIC spectrophotometer. Experiments were conducted with an amount of embryo extract that was in the linear range of the assay and enzyme activity was normalized to protein concentrations in each homogenate measured using the Bradford assay (BioRad). Enzyme activity was calculated using the formula: Units/mg protein = ΔA_340_/minute ÷ 6.22 × mg enzyme/ml reaction mixture, as described by Worthington [89].

For phosphofructokinase assays, we used the Phosphofructokinase activity colorimetric assay kit (BioVision, Milpitas, CA, USA), which converts fructose-6-phosphate and ATP to fructose-diphosphate and ADP. The final product, NADH, reduces a colorless probe to a colored product with strong absorbance at 450 nm. The absorbance was measured with a TECAN INFINITE m200 microplate reader. Experiments were conducted with an amount of embryo extract that was in the linear range of the assay and enzyme activity was normalized to protein concentration.

### Data access

The data reported in this study have been deposited in NCBI’s GEO [90]. The RIP-Chip data are accessible through GEO series accession number GSE49943 and the polysome-microarray data are accessible through GEO series accession number GSE50026.

## Abbreviations

AEBSF: 4-(2-aminoethyl) benzenesulfonyl fluoride hydrochloride; AGO: Argonaute; bp: Base pair; CCT: Chaperonin containing TCP-1; DTT: Dithiothreitol; FDR: False discovery rate; GEO: Gene Expression Omnibus; GO: Gene Ontology; KEGG: Kyoto Encyclopedia of Genes and Genomes; miRNA: MicroRNA; RIP: RNA co-immunoprecipitation; RIP-Chip: RNA co-immunoprecipitations followed by microarray analysis; RMA: Robust Multi-array Average; RT-qPCR: Reverse transcription followed by quantitative polymerase chain reaction; SAM: Significance Analysis of Microarrays; SRE: Smaug recognition element; TI: Translation index; TRiC: TCP1-ring complex; UTR: Untranslated region.

## Competing interests

The authors declare that they have no competing interests.

## Authors’ contributions

HDL and CAS conceived of the project and designed its overall goals. LC and JDL are co-supervised by HDL and CAS; JGD and MHKC by CAS; XL by QM and HDL; ZY and NUS by HDL. All experiments and analyses were carried out by the indicated trainees under the supervision of their respective supervisors. All microarray experiments were conducted with advice of JTW; RIP-Chip and RT-qPCR validation was carried out by LC; polysome gradient-microarrays by JGD; RIP-Chip data analysis by LC with the assistance of XL; polysome gradient-microarray data were normalized by XL and JGD; subsequent polysome gradient-microarray data analyses and SRE-enrichment analyses were carried out by JGD with suggestions from QM; comparisons of binding, translation and stability data sets were carried out by JGD and LC; GO term analysis by LC; generation of the *smaug*^*30*^ and *smaug*^*47*^ alleles by MHKC; mRNA localization enrichment analysis by JDL; enzyme assays by ZY and construction of the *smaug* rescue transgene by NUS. CAS wrote the first draft of the manuscript with input from LC and JGD and the manuscript was revised by CAS and HDL with input from all other authors. All authors read and approved the final manuscript.

## Supplementary Material

Additional file 1 A table listing replicate-to-replicate comparisons of transcript microarray signal intensities from RIP-Chip experiments. Click here for file

Additional file 2 A table listing the genes that encode Smaug-bound mRNAs (that is, genes with an FDR <5% in RIP-Chip experiments). Click here for file

Additional file 3 A figure showing northern analysis of the distribution of 18S ribosomal RNA in polysome gradients run with or without EDTA. Click here for file

Additional file 4 Comparison of wild-type TIs calculated with and without data from pool 2. Click here for file

Additional file 5 **Comparison of the change in TIs in *smaug*-mutant versus wild type with and without data from pool 2.** Click here for file

Additional file 6 **Tables listing the replicate-to-replicate comparisons of transcript microarray signal intensities from wild-type polysome gradients, *smaug*-mutant polysome gradients, and *smaug-*** **mutant polysome gradients with or without puromycin.** Click here for file

Additional file 7 A table listing the genes that encode mRNAs that are translationally repressed by Smaug (that is, genes with an FDR <10% in polysome-gradient microarray experiments). Click here for file

Additional file 8 **A figure comparing the TIs of the bottom 250, 500 and 1,000 Smaug binders in wild-type and *smaug*-mutant embryos.** Click here for file

Additional file 9 **A figure showing the kernel density plots comparing the change in TI in *smaug*-mutant versus wild-type embryos of the top and bottom 250, 500 and 1,000 Smaug binders.** Click here for file

Additional file 10 A figure showing the FDR-based rank of genes from the polysome gradient-microarrays. Click here for file

Additional file 11 The simultaneous overlap between the Smaug-bound mRNAs and those mRNAs that are regulated by Smaug at the level of translational repression and degradation. Click here for file

Additional file 12 Cumulative density plots that show the SRE scores for the 5′ UTR, open reading frame and 3′ UTR of Smaug-target mRNAs that are translationally repressed, degraded and both repressed and degraded. Click here for file

Additional file 13 A table listing the Fly-FISH degradation categories and localization patterns enriched among Smaug-bound mRNAs. Click here for file

Additional file 14 A figure showing the Fly-FISH degradation categories and localization patterns enriched among Smaug-bound mRNAs. Click here for file

Additional file 15 A table listing the genes that encode Smaug-bound mRNAs that are localized to the posterior of the embryo. Click here for file

Additional file 16: A table listing the genes that encode Smaug-bound mRNAs that are annotated as having roles in cell cycle, checkpoint response and/or response to DNA damage. Click here for file

Additional file 17 A table listing the genes that encode Smaug-bound mRNAs annotated as having roles in transcription and/or chromatin. Click here for file

Additional file 18 A table listing the genes that encode Smaug-bound mRNAs encoding proteins in the Chaperonin Cpn60/TCP-1 family. Click here for file

Additional file 19 A table listing the genes that encode Smaug-bound mRNAs encoding proteins found in the proteasome regulatory particle and the ubiquitin proteasome pathway. Click here for file

Additional file 20 A table listing the genes that encode Smaug-bound mRNAs encoding proteins associated with lipid droplets. Click here for file

Additional file 21 A table listing the genes that encode Smaug-bound mRNAs encoding metabolic enzymes. Click here for file

Additional file 22 A table listing the genes that encode Smaug-bound mRNAs encoding enzymes involved in glycolysis and related pathways. Click here for file

Additional file 23 **A northern blot that assesses the integrity of *nanos *** **mRNA in polysome gradient pools.** Click here for file
