# Supplementary material for: Global regulation of mRNA translation and stability in the early Drosophila embryo by the Smaug RNA-binding protein
Source: Genome Biol. 2014 Jan 7;15(1):R4. doi: 10.1186/gb-2014-15-1-r4 (PMC4053848; doi:10.1186/gb-2014-15-1-r4)
Supplement: Additional file 1 — A table listing replicate-to-replicate comparisons of transcript microarray signal intensities from RIP-Chip experiments. [file gb-2014-15-1-r4-S1.pdf]

Additional data file 1: Replicate-to-replicate comparisons of transcript microarray signal intensities from RIP-Chip experiments*

	input	Smaug RIP	control RIP
replicate 1a v. replicate 1b	N/A	0.873	0.865
replicate 1a v. replicate 2	0.960	0.822	0.796
replicate 1a v. replicate 3	0.962	0.776	0.697
replicate 1b v. replicate 2	N/A	0.844	0.810
replicate 1b v. replicate 3	N/A	0.802	0.702
replicate 2 v. replicate 3	0.973	0.802	0.725

*The Pearson correlation coefficient was calculated for each pair of replicates and was used to determine the degree of similarity between them. Replicates 1a and 1b are technical replicates of one another, while replicates 1, 2 and 3 are biological replicates.
