# Supplementary material for: Global regulation of mRNA translation and stability in the early Drosophila embryo by the Smaug RNA-binding protein
Source: Genome Biol. 2014 Jan 7;15(1):R4. doi: 10.1186/gb-2014-15-1-r4 (PMC4053848; doi:10.1186/gb-2014-15-1-r4)
Supplement: Additional file 3 — A figure showing northern analysis of the distribution of 18S ribosomal RNA in polysome gradients run with or without EDTA. [file gb-2014-15-1-r4-S3.pdf]

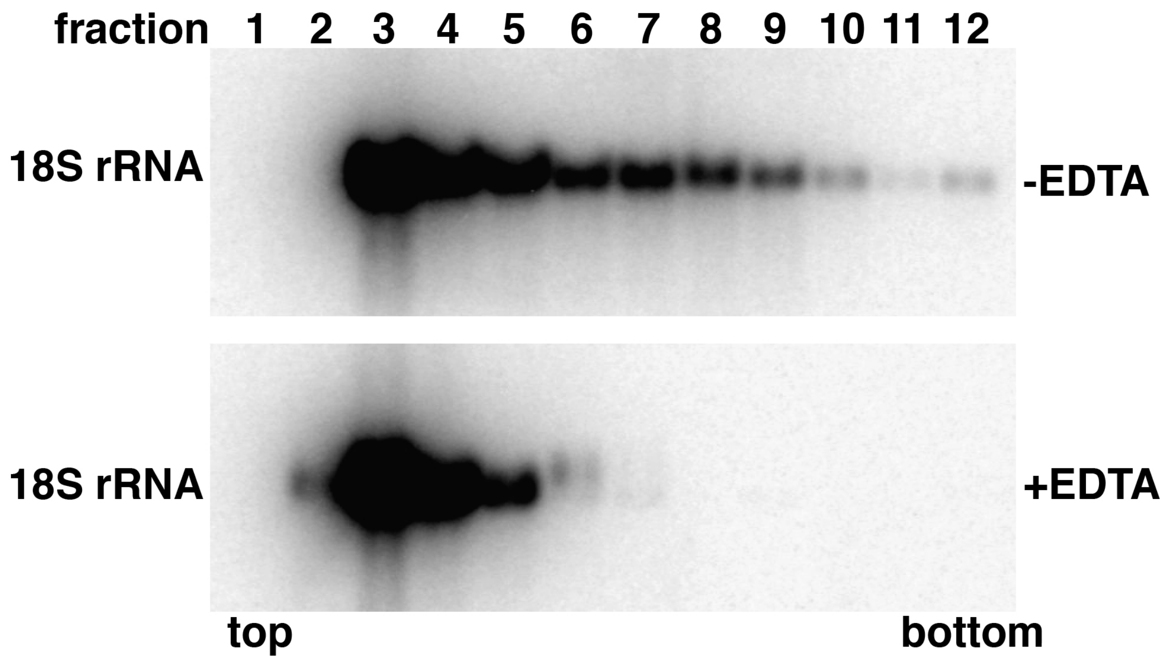

Additional data file 3. Polysome gradient characterization. Polysome gradients were used to fractionate 0-2 hour old embryo extract in the presence or absence of EDTA. Gradients were fractionated into 12 equal fractions, with fraction 1 representing the top of the gradient. RNA harvested from each fraction was analyzed via northern blot using a probe to detect 18S ribosomal RNA.
