# Supplementary material for: Global regulation of mRNA translation and stability in the early Drosophila embryo by the Smaug RNA-binding protein
Source: Genome Biol. 2014 Jan 7;15(1):R4. doi: 10.1186/gb-2014-15-1-r4 (PMC4053848; doi:10.1186/gb-2014-15-1-r4)
Supplement: Additional file 4 — Comparison of wild-type TIs calculated with and without data from pool 2. [file gb-2014-15-1-r4-S4.pdf]

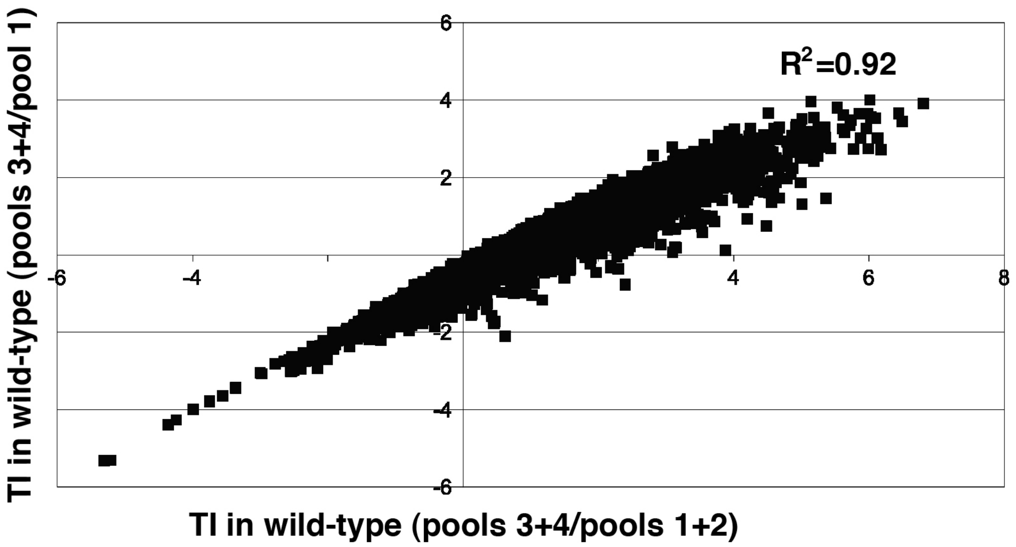

Additional data file 4. Inclusion of Pool 2 in the denominator when calculating the TI does not significantly alter the values calculated without Pool 2. To assess whether including Pool 2 in the calculation had a significant effect on the calculated TIs for mRNAs in wild type embryos, we compared TI = [3 + 4]/[1] on the y-axis to TI = [3 + 4]/[1 + 2] on the x-axis. The fact that the Pearson $R^2 = 0.92$ shows that the inclusion of Pool 2 does not significantly alter the TI calculation used for our analyses, which excluded Pool 2 since it was likely to contain a mix of free and polysome-bound mRNAs.
