# Supplementary material for: Global regulation of mRNA translation and stability in the early Drosophila embryo by the Smaug RNA-binding protein
Source: Genome Biol. 2014 Jan 7;15(1):R4. doi: 10.1186/gb-2014-15-1-r4 (PMC4053848; doi:10.1186/gb-2014-15-1-r4)
Supplement: Additional file 6 — Tables listing the replicate-to-replicate comparisons of transcript microarray signal intensities from wild-type polysome gradients, smaug -mutant polysome gradients, and smaug- mutant polysome gradients with or without puromycin. [file gb-2014-15-1-r4-S6.pdf]

**Additional data file 6:**

**Replicate-to-replicate comparisons of transcript microarray signal intensities from wild-type polysome gradients***

| | fraction 1 | fraction 2 | fraction 3 | fraction 4 |
|---|---|---|---|---|
| replicate 1 v. replicate 2 | 0.51 | 0.69 | 0.69 | 0.83 |
| replicate 1 v. replicate 3 | 0.57 | 0.86 | 0.87 | 0.95 |
| replicate 2 v. replicate 3 | 0.86 | 0.68 | 0.77 | 0.87 |

**Replicate-to-replicate comparisons of transcript microarray signal intensities from *smaug* mutant polysome gradients***

| | fraction 1 | fraction 2 | fraction 3 | fraction 4 |
|---|---|---|---|---|
| replicate 1 v. replicate 2 | 0.67 | 0.77 | 0.79 | 0.70 |
| replicate 1 v. replicate 3 | 0.63 | 0.69 | 0.84 | 0.82 |
| replicate 2 v. replicate 3 | 0.60 | 0.70 | 0.84 | 0.82 |

**Replicate-to-replicate comparisons of transcript microarray signal intensities from *smaug* mutant polysome gradients +/- puromycin***

| | fraction 1 | fraction 2 | fraction 3 | fraction 4 |
|---|---|---|---|---|
| replicate 1 v. replicate 2 (- puromycin) | 0.68 | 0.95 | 0.95 | 0.96 |
| replicate 1 v. replicate 2 (+ puromycin) | 0.90 | 0.96 | 0.96 | 0.74 |

*The Pearson correlation coefficient was calculated for each pair of replicates and was used to determine the degree of similarity between them. All replicates are biological replicates.
