# Supplementary material for: Global regulation of mRNA translation and stability in the early Drosophila embryo by the Smaug RNA-binding protein
Source: Genome Biol. 2014 Jan 7;15(1):R4. doi: 10.1186/gb-2014-15-1-r4 (PMC4053848; doi:10.1186/gb-2014-15-1-r4)
Supplement: Additional file 8 — A figure comparing the TIs of the bottom 250, 500 and 1,000 Smaug binders in wild-type and smaug -mutant embryos. [file gb-2014-15-1-r4-S8.pdf]

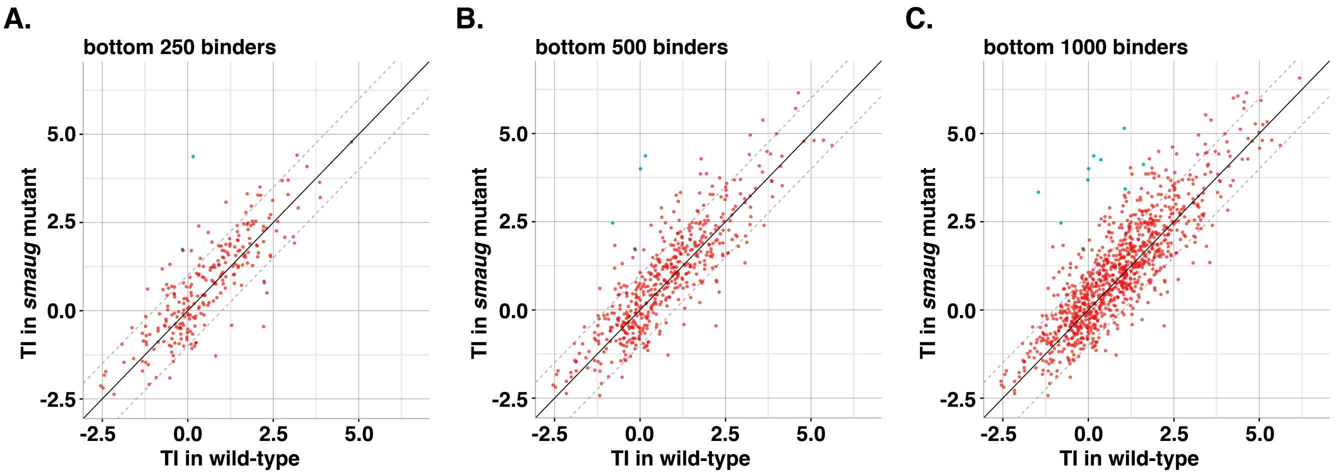


**Additional data file 8. Comparison of the TIs in wild-type versus *smaug*-mutant embryos for mRNAs that are unlikely to be bound by Smaug.** The 250 **(A)**, 500 **(B),** and 1000 **(C)** bottom Smaug binders are the genes whose mRNAs show the lowest fold-enrichment in Smaug RIPs versus control RIPs. The averages, across three biological replicates, of the TI in *smaug* mutants and wild type for each of these groups of genes were plotted against one another. The blue dots represent transcripts that show an increase in TI in *smaug* mutants versus wild type at an FDR of <5%.
