# Supplementary material for: Global regulation of mRNA translation and stability in the early Drosophila embryo by the Smaug RNA-binding protein
Source: Genome Biol. 2014 Jan 7;15(1):R4. doi: 10.1186/gb-2014-15-1-r4 (PMC4053848; doi:10.1186/gb-2014-15-1-r4)
Supplement: Additional file 9 — A figure showing the kernel density plots comparing the change in TI in smaug -mutant versus wild-type embryos of the top and bottom 250, 500 and 1,000 Smaug binders. [file gb-2014-15-1-r4-S9.pdf]

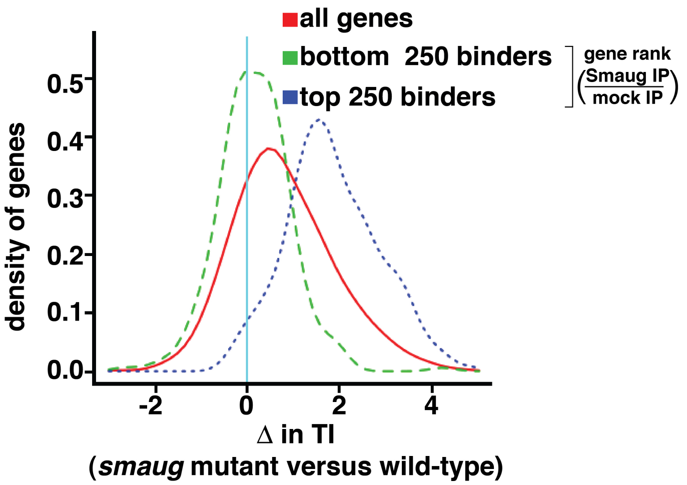

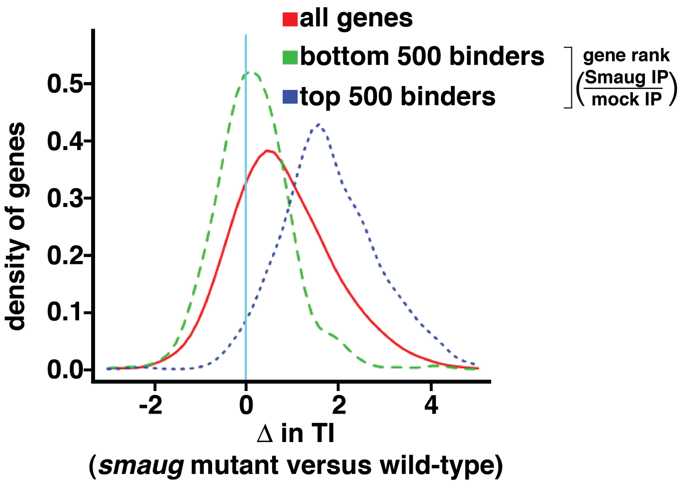

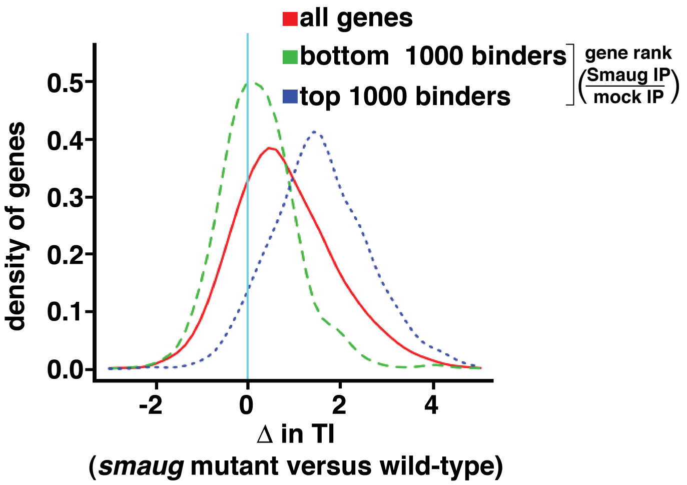

Additional data file 9. Kernel density plots comparing the change in TI in *smaug*-mutant versus wild-type embryos for the top and bottom Smaug binders. Kernel density plots show the change in TI in *smaug*-mutant versus wild-type embryos for the top and bottom 250 **(A),** 500 **(B),** and 1000 **(C)** Smaug binders. These top and bottom binders are the genes whose mRNAs show the highest and lowest fold-enrichment in Smaug RIPs versus control RIPs, respectively. Each graph also contains a plot of all genes in the data set and a light blue vertical line that indicates the position of genes showing no change in their mRNAs' distribution in polysome gradients in *smaug*-mutant compared to wild-type embryos.
