# Supplementary material for: Global regulation of mRNA translation and stability in the early Drosophila embryo by the Smaug RNA-binding protein
Source: Genome Biol. 2014 Jan 7;15(1):R4. doi: 10.1186/gb-2014-15-1-r4 (PMC4053848; doi:10.1186/gb-2014-15-1-r4)
Supplement: Additional file 10 — A figure showing the FDR-based rank of genes from the polysome gradient-microarrays. [file gb-2014-15-1-r4-S10.pdf]

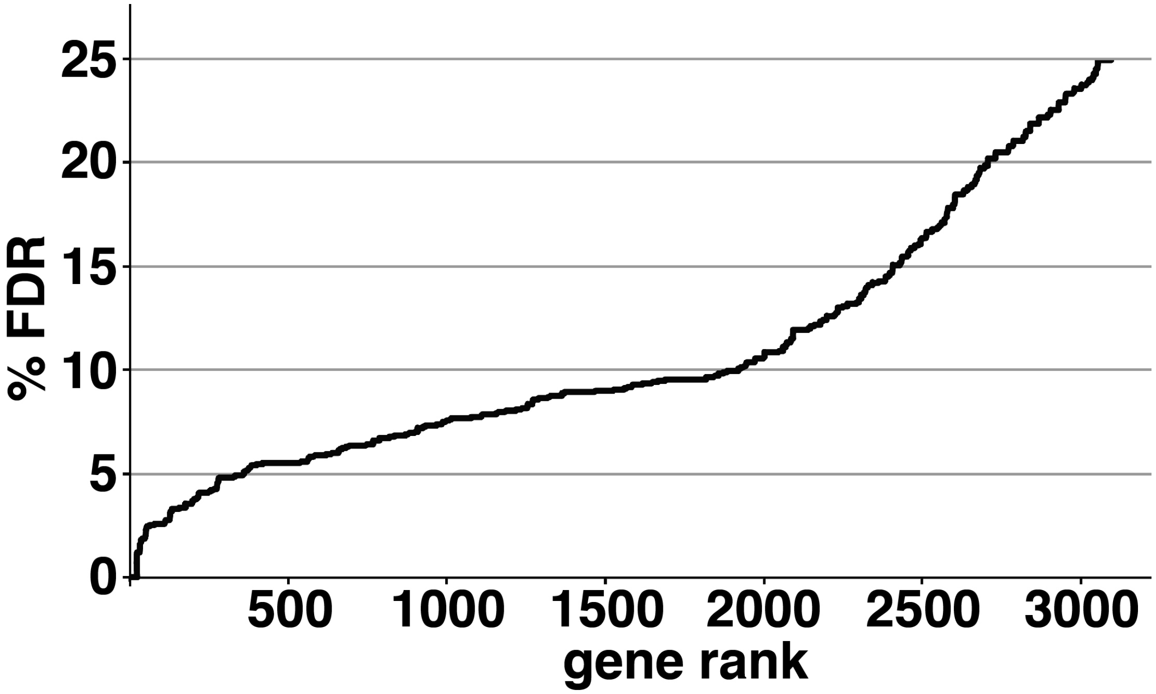

Additional data file 10. FDR-based rank of genes from polysome gradient-microarrays. The FDR of each gene based on its change in TI in *smaug*-mutant versus wild-type embryos was used to rank genes, with the lowest ranking gene having an FDR of 0%. Only genes with an FDR<25% were used in this plot.
