# Supplementary material for: Global regulation of mRNA translation and stability in the early Drosophila embryo by the Smaug RNA-binding protein
Source: Genome Biol. 2014 Jan 7;15(1):R4. doi: 10.1186/gb-2014-15-1-r4 (PMC4053848; doi:10.1186/gb-2014-15-1-r4)
Supplement: Additional file 11 — The simultaneous overlap between the Smaug-bound mRNAs and those mRNAs that are regulated by Smaug at the level of translational repression and degradation. [file gb-2014-15-1-r4-S11.pdf]

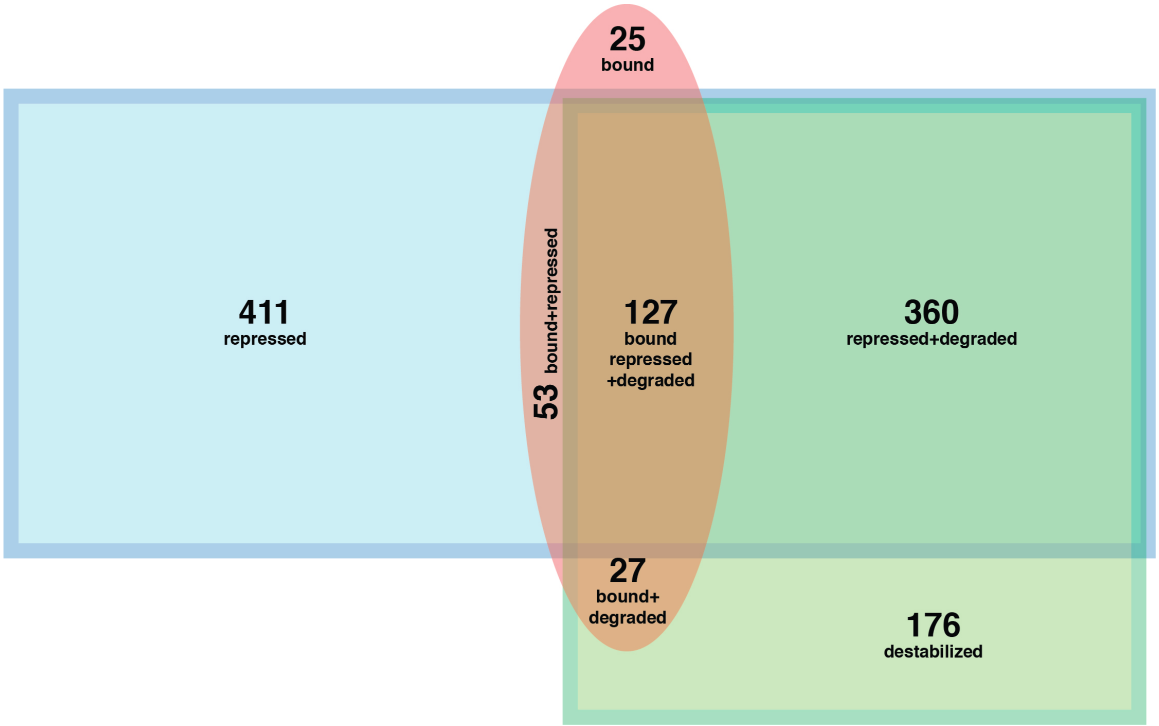

Additional data file 11. Overlaps between Smaug-bound genes and Smaug-regulated genes. Areas of the overlapping regions are approximately proportional to the degree of overlap of the indicated data sets. Note that for this comparison any gene that was not scored as expressed in all three data sets is not included.
