# Supplementary material for: Global regulation of mRNA translation and stability in the early Drosophila embryo by the Smaug RNA-binding protein
Source: Genome Biol. 2014 Jan 7;15(1):R4. doi: 10.1186/gb-2014-15-1-r4 (PMC4053848; doi:10.1186/gb-2014-15-1-r4)
Supplement: Additional file 12 — Cumulative density plots that show the SRE scores for the 5′ UTR, open reading frame and 3′ UTR of Smaug-target mRNAs that are translationally repressed, degraded and both repressed and degraded. [file gb-2014-15-1-r4-S12.pdf]

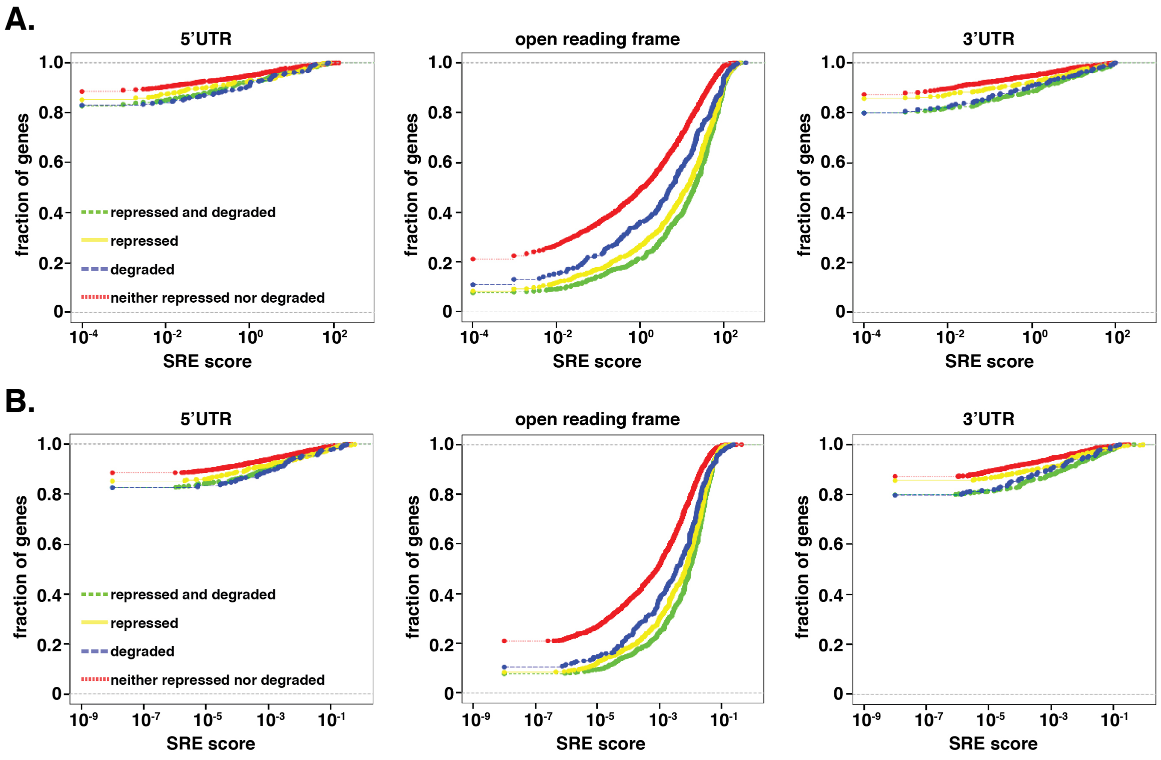

Additional data file 12. SRE scores for the 5'UTR, open reading frame and 3'UTR of Smaug-regulated mRNAs. Cumulative distribution plots show the SRE scores for the indicated region of the mRNA for Smaug-target transcripts that are translationally repressed and degraded by Smaug, those that are just translationally repressed, those that are just degraded, and those that are neither repressed nor degraded by Smaug. **(B)** Shows the results when SRE scores are divided by the length of the relevant region of the transcripts in nucleotides while **(A)** does not take into account feature length.
