# Supplementary material for: Global regulation of mRNA translation and stability in the early Drosophila embryo by the Smaug RNA-binding protein
Source: Genome Biol. 2014 Jan 7;15(1):R4. doi: 10.1186/gb-2014-15-1-r4 (PMC4053848; doi:10.1186/gb-2014-15-1-r4)
Supplement: Additional file 14 — A figure showing the Fly-FISH degradation categories and localization patterns enriched among Smaug-bound mRNAs. [file gb-2014-15-1-r4-S14.pdf]

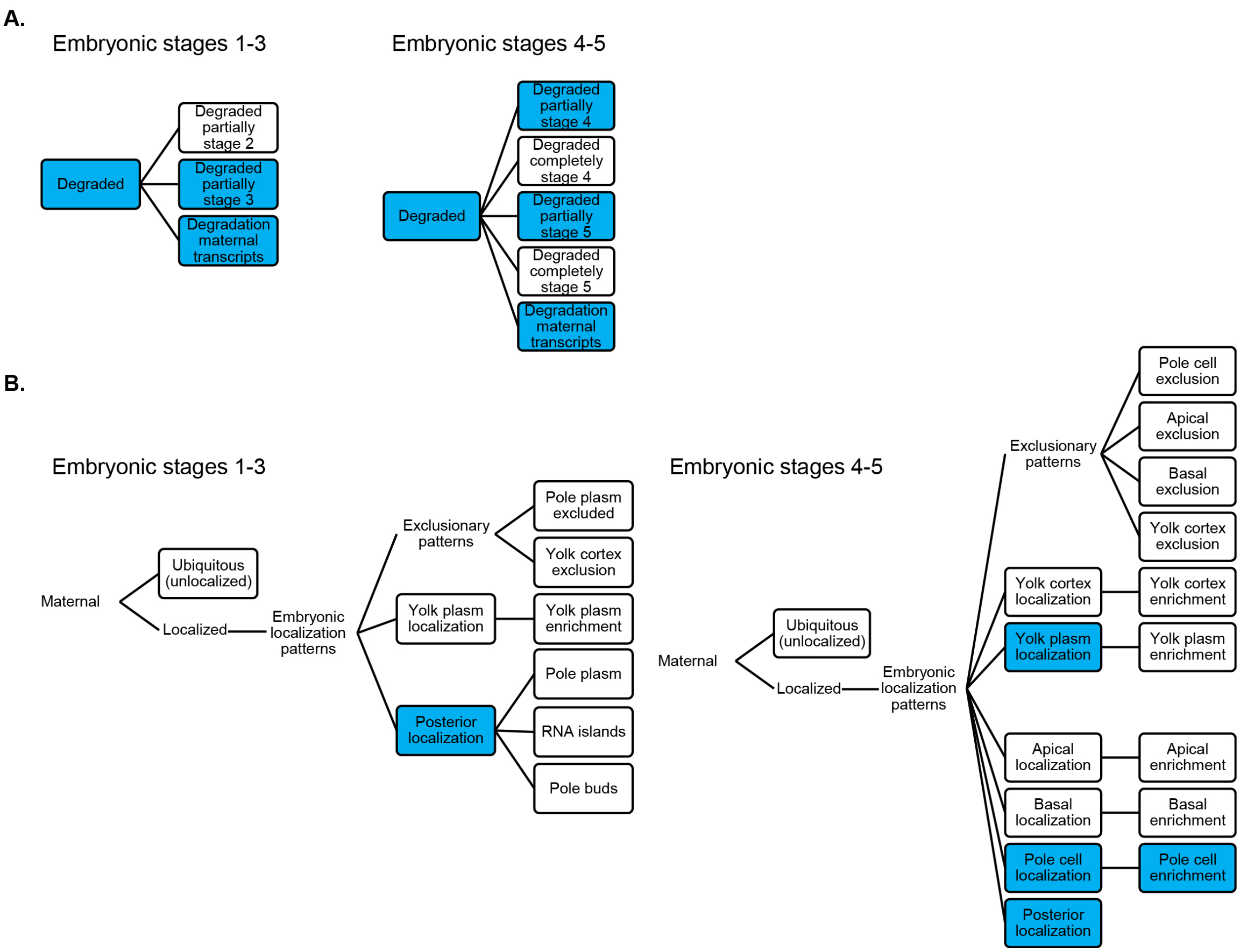

Additional data file 14. Fly-FISH degradation categories and localization patterns enriched among Smaug-bound mRNAs. A search of the Fly-FISH database was performed to identify degradation categories (A) and localization patterns (B) that are enriched among Smaug-bound mRNAs for embryonic stages 1–3 and 4–5. For both (A) and (B), categories in closed boxes were tested for enrichment, and the blue shading indicates the degradation categories and localization patterns that were enriched among the Smaug-bound mRNAs. Detailed results for highlighted categories are presented in Additional data file 9.
