# Supplementary material for: Global regulation of mRNA translation and stability in the early Drosophila embryo by the Smaug RNA-binding protein
Source: Genome Biol. 2014 Jan 7;15(1):R4. doi: 10.1186/gb-2014-15-1-r4 (PMC4053848; doi:10.1186/gb-2014-15-1-r4)
Supplement: Additional file 23 — A northern blot that assesses the integrity of nanos mRNA in polysome gradient pools. [file gb-2014-15-1-r4-S23.pdf]

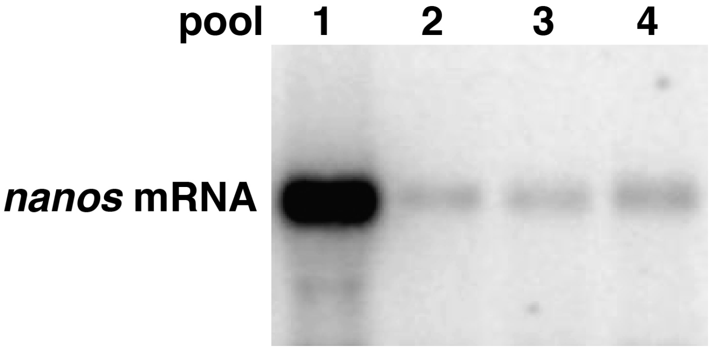

Additional data file 23. Assessing the integrity of polysome fractionated mRNA. Aliquots of RNA from polysome gradient pools 1, 2, 3 and 4 that were analyzed by microarray were subjected to northern blot analysis by probing for *nanos* mRNA.
